# Natural products as kinase inhibitors in lung cancer: molecular mechanisms, therapeutic potential, and clinical trials

**DOI:** 10.3389/fphar.2026.1764550

**Published:** 2026-02-25

**Authors:** Adil Farooq Wali, Sirajunisa Talath, Rasha Babiker, Mohamed El-Tanani, Imran Rashid Rangraze, Walaa Ibraheem, Yusra Al Aldhaheri, Shakta Mani Satyam, Yahia El-Tanani

**Affiliations:** 1 RAK College of Pharmacy, RAK Medical and Health Science University, Ras Al Khaimah, United Arab Emirates; 2 RAK College of Medical Sciences, RAK Medical and Health Sciences University, Ras-al-Khaimah, United Arab Emirates; 3 Department of Biology, College of Science, United Arab Emirates University, Al Ain, United Arab Emirates; 4 Royal Cornwall Hospital Trust, NHS, Bath, United Kingdom

**Keywords:** bioavailability enhancement, clinical trials, natural kinase inhibitors, non-small cell lung cancer, off-target effects, tyrosine kinase inhibitors

## Abstract

Lung cancer remains a leading cause of cancer mortality worldwide, with current treatments often limited by toxicity and resistance. Dysregulated kinase signaling particularly involving EGFR, PI3K/AKT/mTOR, MAPK, and ALK pathways drives tumor growth, survival, and metastasis. While synthetic kinase inhibitors have improved outcomes, their use is constrained by adverse effects and acquired resistance. Natural kinase inhibitors (NKIs) derived from plants, marine organisms, and microorganisms offer a promising alternative due to their multi-targeted action, lower toxicity, and potential to overcome resistance. This review aims to evaluate the molecular mechanisms, therapeutic potential, and clinical relevance of NKIs in lung cancer management. Key compounds such as curcumin, resveratrol, quercetin, genistein, and epigallocatechin gallate inhibit critical kinases, modulating pathways that regulate proliferation, apoptosis, angiogenesis, and metastasis. Preclinical studies demonstrate significant anticancer activity, while emerging clinical evidence supports their role as adjuncts or alternatives to conventional therapies. Strategies such as nanotechnology-based delivery systems and combination regimens further enhance bioavailability and efficacy. Despite these advantages, challenges persist, including poor solubility, rapid metabolism, and limited clinical validation. Future research should focus on optimizing formulations, elucidating pharmacokinetics, and conducting large-scale clinical trials to confirm safety and effectiveness. Integration of NKIs into personalized treatment paradigms could transform lung cancer therapy, offering cost-effective, less toxic, and multi-targeted approaches to improve patient outcomes.

## 1 Introduction

### 1.1 Overview

Lung carcinoma is a type of cancer that begins in the tissues of the lungs, most often in the cells lining the airways. It comprises two categories: Non-Small Cell Lung Cancer (NSCLC), accounting for 85% of cases (Oh et al., 2024), and Small Cell Lung Cancer (SCLC), a more aggressive form accounting for 15% (Sher et al., 2008; Zappa and Mousa, 2016; Pothongsrisit and Pongrakhananon, 2021). Smoking is the main risk; exposure to asbestos, radon gas, second-hand smoke, a family history of lung cancer, and carcinogens also significantly increases risk (Hecht, 1996; Whitrow et al., 2003; Stayner et al., 2008; Thompson, 2014; Benusiglio et al., 2021; Bittoni et al., 2024). Common symptoms include shortness of breath, chest discomfort, unexplained weight loss, chest pain, and persistent coughing that may produce blood. (Thompson, 2014; Larsen and Minna, 2011). Diagnostic methods encompass imaging tests like CT scans and chest X-rays, biopsies, molecular testing and sputum cytology to identify specific genetic mutations (Patharia et al., 2024). Staging of lung cancer, ranging from stage I (localized) to stage IV (metastasized), guides therapeutic choices and may include chemotherapy, radiation therapy, immunotherapy, surgery, and targeted therapy (Rami-Porta et al., 2018; Ahmad et al., 2020; Kim et al., 2022; Daly et al., 2022). The prognosis depends heavily on the cancer’s stage at diagnosis, with early detection being crucial for better outcomes (Fitzgerald et al., 2022; Hawkes, 2019; Smith et al., 2002). Prevention strategies focus on smoking cessation, avoiding second-hand smoke, testing homes for radon, ensuring occupational safety, and maintaining a healthy lifestyle (Hassanein, 2023). Advances in research are continually improving treatment approaches and early detection techniques, offering hope for better survival rates and personalized medicine tailored to individual patient profiles (Sherani et al., 2024).

### 1.2 Prevalence and significance of lung cancer

Considering the data from the World Cancer Research Fund International, lung cancer continues to be a major worldwide health concern. In 2022, the worldwide incidence of lung cancer was reported at 2,480,675 cases, with an age-standardized rate (ASR) of 23.6 per 100,000 individuals. China possessed the greater prevalence of lung cancer cases, with 1,060,584 cases and an ASR of 40.8 per 100,000. The United States followed with 226,033 cases (ASR of 31.9), and Japan ranked third with 136,723 cases (ASR of 30.5). Other countries with high lung cancer incidence included India (81,748 cases, ASR 5.8), the Russian Federation (70,362 cases, ASR 26.0), Germany (62,025 cases, ASR 28.1), the United Kingdom (50,700 cases, ASR 30.1), France (49,613 cases, ASR 35.9), Brazil (44,213 cases, ASR 14.6), and Italy (43,808 cases, ASR 24.6). The data showed a higher incidence in men compared to women. Globally, men accounted for 1,572,045 cases with an ASR of 31.1 per 100,000, while women had 908,630 cases with an ASR of 16.2 per 100,000. China again led in both categories, with 658,722 cases (ASR 52.0) in men and 401,862 cases (ASR 30.3) in women. The United States had 112,343 cases (ASR 39.9) in men and 113,690 cases (ASR 14.7) in women, highlighting the significant gender disparity in lung cancer incidence. In terms of mortality, lung cancer caused 1,817,469 deaths globally in 2022, with an ASR of 16.8 per 100,000. China has experienced increased mortality related to lung cancer deaths, with 733,291 deaths (ASR 26.7). The United States followed with 127,693 deaths (ASR 16.6), and Japan had 83,243 deaths (ASR 14.2). Other countries with high lung cancer mortality included India (75,031 deaths, ASR 5.3), the Russian Federation (51,887 deaths, ASR 21.4), Germany (47,731 deaths, ASR 21.4), Turkey (38,505 deaths, ASR 35.1), Brazil (38,292 deaths, ASR 12.5), France (36,876 deaths, ASR 24.2), and Italy (35,668 deaths, ASR 18.0) (Siegel et al., 2025; Casolino et al., 2024; Chidebe et al., 2025; Luo et al., 2023).

### 1.3 Current treatment modalities and their limitations

Although lung cancer therapy has advanced considerably, each treatment modality continues to present inherent limitations (Bertolaccini et al., 2024a; Sharma et al., 2022a). Surgery is effective for early-stage cancer, removing localized tumors through procedures like lobectomy and pneumonectomy. However, it's unsuitable for advanced stages or patients with significant health issues, and there is a risk of recurrence and complications like infections and reduced lung function (Bertolaccini et al., 2024b). When radiation treatment is combined with surgeries or utilized independently, it targets cancer cells with high-energy rays but can damage surrounding healthy tissues, causing side effects like fatigue and pneumonitis (Li et al., 2025). Some cancer cells also develop resistance, and the treatment is less effective for widespread metastasis. Chemotherapy employs medications to destroy cells divide quickly, used before or after surgery, or as the basic therapy in advanced stages. Its non-selectivity causes severe side effects, including nausea, fatigue, and immune suppression, and cancer cells can develop resistance. Focused treatment targets certain genetic alterations in cancerous cells, like Epidermal Growth Factor Receptor (EGFR) and ALK (anaplastic lymphoma kinase) inhibitors (Weiss et al., 2022). While effective for some, cancer can develop resistance, and side effects include rash and liver problems. Immunotherapy enhances immunological system’s capacity to fight cancer, but not all patients respond, and side effects can include inflammation of healthy tissues (Anand et al., 2023). Combination therapy, using multiple treatments, aims to increase efficacy but also raises the risk of toxicity and side effects (Malik et al., 2020). Emerging treatments like CAR-T cell therapy and oncolytic virus therapy are promising but still experimental, with unknown long-term effects and limited availability (Zarezadeh Mehrabadi et al., 2022). The creation of medications with great selectivity and efficacy to operate against a particular target has been a major focus of anticancer drug research. Clinical experience, particularly the identification of cancer medication resistance, has shown, even in cases when the target is blocked or inactivated, single targeting may not always result in the intended biological impact (Liu et al., 2024; Adhikari et al., 2024). The cause is resistance arises because of a malignant cell either adopting new growth and multiplication routes or self-modifying the target through mutation. Targeting a single oncoprotein may not yield a favorable therapeutic outcome and is often insufficient to achieve durable remission in patients (Wang et al., 2019; Lei et al., 2023). Thus, modifying the biological network has advantages. Diverse cell populations with a range of genetic and phenotypic traits frequently make up tumors. This heterogeneity can be addressed by multi-target medications and act on several cancer-driving pathways at once (Aranda-Anzaldo and Dent, 2020).

### 1.4 Role of kinase inhibitors in cancer treatment

Kinase inhibitors are essential in cancer treatment by targeting specific enzymes known as kinases and are engaged in controlling a number of biological functions, like cell division, proliferation, and survival. These enzymes can become dysregulated in cancer, resulting in unchecked cell division and tumor growth. Kinase inhibitors work by blocking the activity of these enzymes and interrupting the signaling pathways drive cancer progression (Xu et al., 2025).

### 1.5 Mechanism of action of kinase inhibitors

**FIGURE 1:**
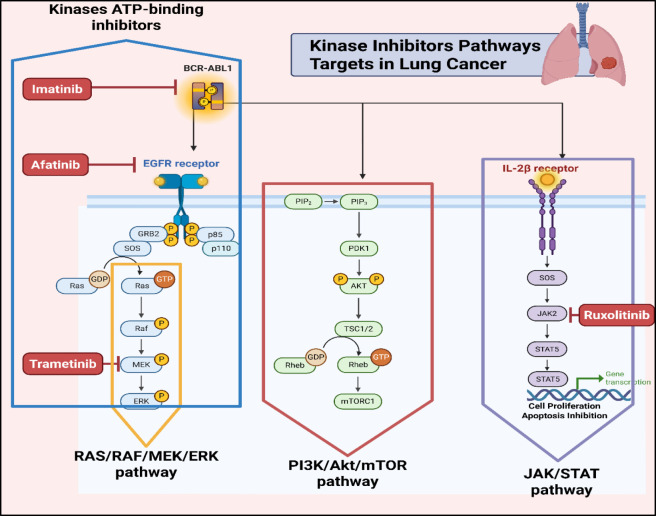
Illustrates the major kinase signaling pathways involved in the pathogenesis and progression of Non-Small Cell Lung Cancer (NSCLC) and their potential therapeutic targeting using synthetic kinase inhibitors. Three critical signaling cascades are depicted: The RAS/RAF/MEK/ERK pathway (Rat Sarcoma/Rapidly Accelerated Fibrosarcoma/Mitogen-Activated Protein Kinase/Extracellular Signal-Regulated Kinase), which is activated downstream of the Epidermal Growth Factor Receptor (EGFR) and regulates cell proliferation and differentiation. This pathway can be inhibited by drugs like Trametinib and Afatinib, which target MEK and EGFR, respectively; PI3K/AKT/mTOR pathway (Phosphoinositide 3-Kinase/Protein Kinase B/Mammalian Target of Rapamycin), which controls cell growth, metabolism, and survival. This pathway is activated by EGFR via PI3K, leading to AKT phosphorylation and mTORC1 activation. Although no inhibitor is labeled here, this pathway represents a common target in NSCLC therapy. JAK/STAT pathway (Janus Kinase/Signal Transducer and Activator of Transcription), activated by cytokine receptors such as the interleukin-2 beta (IL-2β) receptor. This signaling promotes gene transcription related to cell survival and proliferation. Ruxolitinib, a JAK inhibitor, blocks this pathway to prevent tumor growth. Kinase inhibitors like Imatinib, Afatinib, Trametinib, and Ruxolitinib exert their therapeutic effects by blocking ATP-binding sites of kinases, thereby interfering with aberrant signaling that drives tumor development in NSCLC. These inhibitors are promising agents in targeted lung cancer therapies aimed at improving patient outcomes. Created in https://BioRender.com Abbreviations: AKT, protein kinase B; EGFR, epidermal growth factor receptor; ERK, extracellular signal-regulated kinase; IL-2β, interleukin-2 beta; JAK, Janus kinase; MEK, mitogen-activated protein kinase; NSCLC, non-small cell lung cancer; PI3K, phosphoinositide 3-kinase; RAF, rapidly accelerated fibrosarcoma; RAS, rat sarcoma; STAT, signal transducer and activator of transcription; mTOR, mechanistic target of rapamycin; mTORC1, mechanistic target of rapamycin complex 1.

Many kinase inhibitors are ATP-competitive as they attach themselves to the kinase’s ATP-binding site, blocking the attachment of ATP and thus preventing the phosphorylation of substrates (Juan et al., 2025). For instance, Imatinib (Gleevec) attaches to the ATP-binding site of the BCR-ABL fusion protein in chronic myeloid leukemia (CML), inhibiting its activity. Allosteric inhibitors bind to regions distinct from the ATP-binding pocket, inducing conformational alterations that diminish kinase catalytic activity (Fang et al., 2025). Trametinib, an allosteric inhibitor of MEK1/2, exemplifies this mechanism (Zhao et al., 2024). Some kinase inhibitors are irreversible, interacting with the kinase via covalent bonds to cause permanent deactivation, like Afatinib, an irreversible EGFR inhibitor used in NSCLC (Cai et al., 2023). Inhibiting kinases disrupt critical signaling pathways vital for the viability and growth of cancer cells. Kinase inhibitors, for instance, can target the PI3K/AKT/mTOR pathway and regulates cell survival and proliferation, to cause apoptosis and slow tumour development (Tufail et al., 2024). Similarly, the RAS/RAF/MEK/ERK pathway, engaged in the development and proliferation of cells, can be inhibited to halt cancer cell proliferation (Barbosa et al., 2021). Signals are sent to the nucleus via cytokine receptors via the JAK/STAT pathway and influences gene expression, is another target, with inhibitors like Ruxolitinib disrupting this pathway and promoting cancer cell death (Hu et al., 2021) (Figure 1). As illustrated in Figure 1, major kinase signaling pathways like RAS/RAF/MEK/ERK, PI3K/AKT/mTOR, and JAK/STAT drive NSCLC pathogenesis, underscoring potential targets for synthetic inhibitors.

### 1.6 Targeting kinase signalling pathways in non-small cell lung cancer (NSCLC) therapy

### 1.7 Importance in targeted cancer therapy

Among most benefits of kinase inhibitors, is the ability to specifically target genetic mutations are common in lung cancer. For instance, changes to the EGFR are found in a substantial proportion of NSCLC cases (Shah and Lester, 2020; Cooper et al., 2022). Kinase inhibitors target these mutations, leading to the inhibition of the aberrant signaling pathways promote cancer cell proliferation and survival. By directly targeting the mutations, these drugs can effectively halt the progression of the disease with greater precision than traditional treatments (Tomuleasa et al., 2024; Manzari et al., 2021). For example, NSCLC patients harboring EGFR mutations treated with EGFR inhibitors typically experience better clinical outcomes, like reduced tumor size and extended periods without disease progression. Similarly, ALK inhibitors like Crizotinib (Xalkori) and Alectinib (Alecensa) have shown remarkable effectiveness in patients with ALK-positive NSCLC, leading to substantial improvements in survival rates (Carcereny et al., 2021; Yu et al., 2019).

### 1.8 Scope and objectives of the review

The present review addresses the origins, mechanisms, therapeutic advantages, and difficulties of natural kinase inhibitors (NKIs), with an emphasis on their application in lung cancer treatment. It aims to define NKIs, elucidate their biochemical mechanisms, identify key sources from plants, marine organisms, and microorganisms, and evaluate their clinical advantages like reduced side effects and multi-targeted action. The review addresses the limitations and challenges of NKIs, including bioavailability and standardization issues, and summarizes recent research and clinical studies demonstrating their efficacy and safety. Future directions are highlighted, focusing on improved formulations, combination therapies, and the identification of new targets, with the ultimate goal of promoting awareness and integration of NKIs into mainstream cancer treatment to enhance patient outcomes.

### 1.9 Methodology of literature search

This review was conducted following a structured and systematic literature search strategy to identify relevant studies on natural kinase inhibitors in lung cancer. Electronic databases including PubMed, Scopus, Web of Science, and Google Scholar were searched for peer-reviewed articles published primarily between 2000 and 2025, with emphasis on recent and clinically relevant studies. The search strategy employed a combination of Medical Subject Headings (MeSH) terms and free-text keywords, including but not limited to: *“natural kinase inhibitors,” “plant-derived kinase inhibitors,” “natural products,” “lung cancer,” “non-small cell lung cancer,” “EGFR,” “PI3K/AKT/mTOR,” “MAPK,” “JAK/STAT,” “clinical trials,” and “targeted therapy.”* Boolean operators (AND/OR) were used to refine the search results.

Inclusion criteria encompassed original research articles, clinical trials, systematic reviews, and relevant preclinical studies that investigated the molecular mechanisms, therapeutic potential, or clinical relevance of natural kinase inhibitors in lung cancer or related solid tumors. Studies focusing exclusively on synthetic kinase inhibitors without translational relevance to natural compounds were excluded unless used for comparative or contextual purposes. Non-English publications, conference abstracts without full text, and studies lacking sufficient methodological detail were also excluded.

## 2 Molecular pathways and targets in lung cancer

Numerous important signaling pathways control cellular functions including growth, division, survival, as well as death are involved in the initiation and spread of lung cancer. The major signaling pathways implicated in lung cancer include.

### 2.1 EGFR pathway

**FIGURE 2:**
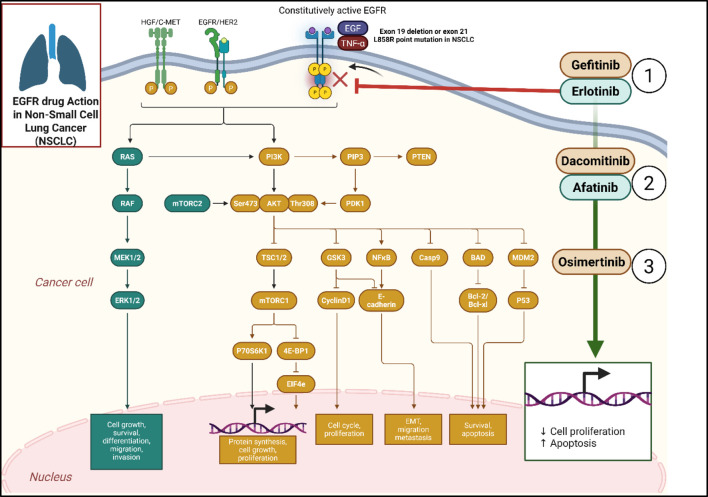
Illustrates the molecular mechanisms and downstream signaling pathways influenced by epidermal growth factor receptor (EGFR) mutations in Non-Small Cell Lung Cancer (NSCLC) and the action of EGFR-targeting drugs. Activating EGFR mutations, such as exon 19 deletion or exon 21 L858R point mutation, lead to constitutive activation of EGFR and stimulation of downstream pathways including RAS/RAF/MEK/ERK and PI3K/AKT/mTOR, promoting cancer cell proliferation, survival, migration, and metastasis. (1) First-generation EGFR inhibitors like Gefitinib and Erlotinib block EGFR signaling at the receptor level. (2) Second-generation inhibitors Afatinib and Dacomitinib provide broader and irreversible EGFR inhibition. (3) Third-generation inhibitor Osimertinib effectively targets both activating EGFR mutations and the T790M resistance mutation, leading to decreased cell proliferation and increased apoptosis. These drugs modulate multiple intracellular processes including cell cycle progression, epithelial-to-mesenchymal transition (EMT), apoptosis regulation, and protein synthesis, contributing to the inhibition of tumor growth in EGFR-mutant NSCLC. Created in https://BioRender.com Abbreviations: AKT, protein kinase B; EGFR, epidermal growth factor receptor; EMT, epithelial-to-mesenchymal transition; ERK, extracellular signal-regulated kinase; MEK, mitogen-activated protein kinase; NSCLC, non-small cell lung cancer; PI3K, phosphoinositide 3-kinase; RAF, rapidly accelerated fibrosarcoma; RAS, rat sarcoma; mTOR, mechanistic target of rapamycin.

One of the most important signaling routes is the EGFR pathway contributes to the growth and progression of NSCLC Figure 2. Changes within the EGFR gene are common in NSCLC, particularly among non-smokers and East Asian populations. The receptor is frequently constitutively activated by these mutations, regardless of ligand binding, promoting the ongoing survival and growth of cells. EGFR is a transmembrane receptor tyrosine kinase, when activated, triggers a cascade of downstream signaling events regulate cellular proliferation, survival, and differentiation (Mitsudomi, 2014; Lee et al., 2024). The L858R point mutation in exon 21 and exon 19 deletions are the most prevalent EGFR mutations (Batra et al., 2023). These mutations enhance the kinase activity of EGFR, promoting oncogenic signaling. EGFR activation begins with the process by which its ligands, including transforming growth factor-alpha (TGF-α) including epidermal growth factor (EGF), attach to the receptor’s outer region. Due to this interaction, tyrosine residues in the receptor’s intracellular domain undergo autophosphorylation and dimerization. The signal is spread downstream by an array of adaptor enzymes and proteins interact with these phosphorylated residues of tyrosine. Activation of EGFR causes downstream signaling from the RAS/RAF/MEK/ERK and PI3K/AKT/mTOR pathways, promoting cancer cell division and survival (Lindsey and Langhans, 2015; Chen et al., 2016). Gefitinib (Iressa) and Erlotinib (Tarceva) are first-generation EGFR tyrosine kinase inhibitors (TKIs) have demonstrated notable effectiveness in treating NSCLC patients with EGFR mutations. They are effective in inducing enhancing progression-free survival and tumour regression (Karachaliou et al., 2019). Dacomitinib and Afatinib (Gilotrif) are second-generation irreversible TKIs bind covalently to the EGFR tyrosine kinase domain, providing a more sustained inhibition of the receptor. Osimertinib (Tagrisso) is a third-generation TKI designed to target both sensitizing EGFR mutations and the T790M resistance mutation and often emerges after first-generation TKIs. Although EGFR TKIs are initially effective, most individuals eventually develop resistance (Șandor et al., 2023). The T790M mutation in the EGFR gene is the most frequent mechanism of resistance to first-generation TKIs. Activation of bypass signaling pathways, like the MET or HER2 pathways, can also confer resistance (Leonetti et al., 2019). In some cases, the tumor may undergo transformation to SCLC and is less responsive to EGFR TKIs (Mambetsariev et al., 2022).

### 2.2 Mechanism of EGFR-Targeted drugs in non-small cell lung cancer (NSCLC)

### 2.3 RAS/RAF/MEK/ERK pathway

**FIGURE 3:**
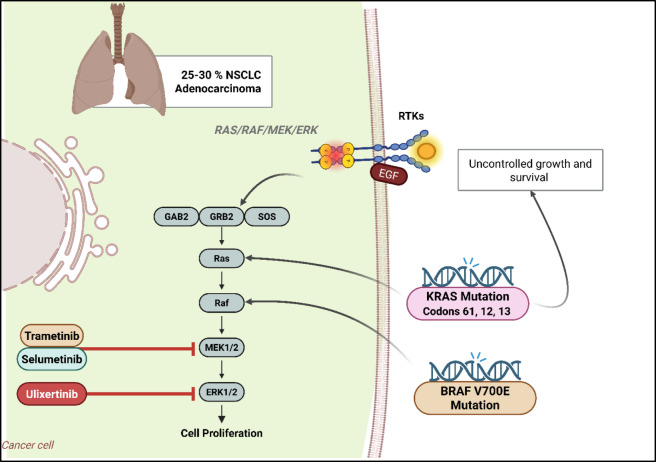
Highlights the RAS/RAF/MEK/ERK signaling cascade in Non-Small Cell Lung Cancer (NSCLC) adenocarcinoma, which is activated in 25%–30% of cases. Binding of epidermal growth factor (EGF) to receptor tyrosine kinases (RTKs) triggers a cascade involving adaptor proteins GAB2, GRB2, and SOS, leading to activation of Ras, Raf, MEK1/2, and ERK1/2. Mutations in KRAS (codons 12, 13, 61) and BRAF V600E lead to persistent pathway activation, resulting in uncontrolled cell proliferation, growth, and survival. Targeted therapies are shown at key steps: Trametinib and Selumetinib inhibit MEK1/2, while Ulixertinib targets ERK1/2, aiming to suppress downstream oncogenic signaling and inhibit tumor progression in NSCLC. Created in https://BioRender.com Abbreviations: BRAF, v-raf murine sarcoma viral oncogene homolog B1; ERK, extracellular signal-regulated kinase; KRAS, Kirsten rat sarcoma viral oncogene homolog; MEK, mitogen-activated protein kinase; NSCLC, non-small cell lung cancer; RAF, rapidly accelerated fibrosarcoma; RAS, rat sarcoma.

The RAS/RAF/MEK/ERK pathway, also known as the MAPK (mitogen-activated protein kinase) pathway, is a critical signaling cascade involved in regulating cell growth, division, differentiation, as well as survival (Baha et al., 2023). This pathway is frequently dysregulated in cancer, including NSCLC (Figure 3). As illustrated in Figure 3, KRAS and BRAF mutations converge on MEK/ERK activation, highlighting why MEK inhibitors may synergize with therapies targeting upstream RTKs in NSCLC. Growth factors, like EGF, usually attach to receptor tyrosine kinases (RTKs), like EGFR, on the cell surface to start the cascade (Hsu and Hung, 2016). Certain tyrosine residues within the intracellular region of the receptor undergo autophosphorylation and receptor dimerization This interaction. Phosphorylated tyrosine residues on RTKs function as adaptor protein docking sites, like Grb2 (Batool et al., 2023). SOS, is a guanine nucleotide exchange factor Grb2 in turn brings to the membrane. SOS activates RAS by facilitating the small GTPase’s ability to substitute GDP with GTP within the RAS proteins. Active RAS-GTP binds to and activates RAF kinases, primarily BRAF and CRAF (Bandaru et al., 2019). This attachment causes RAF dimerization and activation through phosphorylation. Active RAF then phosphorylates and activates MEK (MAPK/ERK kinase). MEK is a dual-specificity kinase phosphorylates ERK (extracellular signal-regulated kinase) on both tyrosine and threonine residues, leading to its activation (Huang et al., 2023). Phosphorylated ERK translocates to the nucleus, where it phosphorylates various transcription factors, like ELK1 and c-Fos. The synthesis of genes associated with the development of cell cycles, growth, division, and viability is regulated by these transcription factors (Ram A. et al., 2023).

A common oncogenic driver in lung cancer is mutations in the RAS gene, namely, KRAS. KRAS mutations occur in around 25%–30% of instances of NSCLC, especially in adenocarcinomas. The most frequent mutations are found in codons 61, 12, and 13 resulting in the RAS protein being persistently activated continuously signals downstream effectors, driving uncontrolled growth and survival of cells (Sinha et al., 2024; Kumar and Kumar, 2022).

Although less common than RAS mutations, mutations in BRAF can also occur in lung cancer. Most prominent mutation, BRAF V600E, causes continuous stimulation of RAF kinase and in turn activates the MEK/ERK cascade (Cicenas et al., 2017; Yan et al., 2022). The continuous activation of the RAS/RAF/MEK/ERK pathway promotes oncogenesis by increasing cell development. It contributes to the resistance mechanisms against various therapies, including chemotherapy and targeted treatments. MEK inhibitors, like Trametinib and Selumetinib, block the activity of MEK1/2 and preventing ERK activation (Ram T. et al., 2023; Wu and Park, 2015). These inhibitors have shown efficacy in tumors with BRAF mutations and are being explored in combination with other therapies to treat KRAS-mutant lung cancers (Jiang L. et al., 2024). Direct ERK inhibitors, like Ulixertinib, are also under investigation. By targeting ERK, these inhibitors aim to block the final step of the pathway, potentially overcoming resistance mechanisms arise from upstream mutations (Kun et al., 2021).

### 2.4 The RAS/RAF/MEK/ERK pathway in NSCLC adenocarcinoma: Mutations and inhibitors

### 2.5 PI3K/AKT/mTOR pathway

**FIGURE 4:**
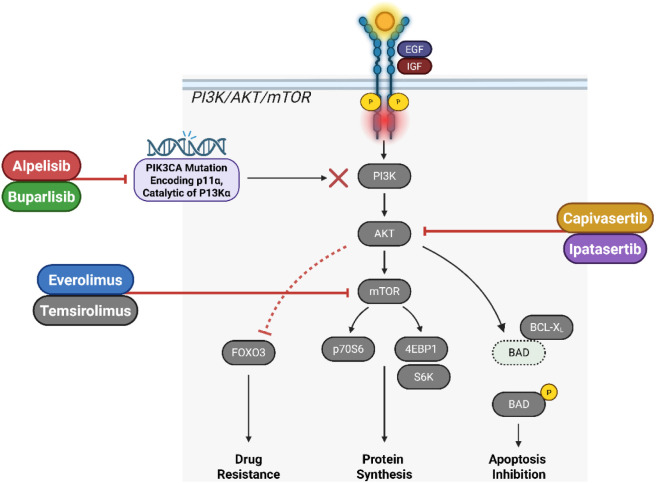
Illustrates the PI3K/AKT/mTOR signaling pathway and its therapeutic inhibition in cancer, particularly in Lung Cancer harbouring PIK3CA mutations (encoding the p110α catalytic subunit of PI3Kα). Activation of this pathway via growth factors such as EGF (Epidermal Growth Factor) and IGF (Insulin-like Growth Factor) promotes tumor cell survival, proliferation, and drug resistance. Key nodes are targeted by specific inhibitors: Alpelisib and Buparlisib inhibit PI3K activity; Capivasertib and Ipatasertib block AKT; and Everolimus and Temsirolimus inhibit mTOR function. Downstream effects include reduced phosphorylation of BAD (a pro-apoptotic protein), thereby inhibiting apoptosis, and modulation of p70S6, 4EBP1, and S6K to affect protein synthesis. Inhibition of mTOR also impacts FOXO3 activity, influencing drug resistance. Collectively, these inhibitors disrupt critical signaling events driving cancer progression, offering potential for targeted therapeutic strategies. Created in https://BioRender.com Abbreviations: AKT, protein kinase B; FOXO3, forkhead box O3; IGF, insulin-like growth factor; PI3K, phosphoinositide 3-kinase; mTOR, mechanistic target of rapamycin.

The PI3K/AKT/mTOR pathway is a prominent regulator of the growth, metabolism, and survival of cells (Figure 4). As illustrated in Figure 4, PI3K/AKT/mTOR pathway activation via PIK3CA mutations promotes tumor survival and resistance, emphasizing inhibitors like Alpelisib and Everolimus as key therapeutic nodes. Aberrations in this pathway, like mutations in PIK3CA or loss of PTEN, contribute to lung cancer progression (Krencz et al., 2021). The pathway is typically initiated via growth factor binding, like insulin-like growth factor (IGF) or EGF, to RTKs on the cell surface. Certain tyrosine residues in the intracellular region of the receptor undergo autophosphorylation or receptor dimerization This interaction. Phosphatidylinositol 3-kinase (PI3K) is recruited to the phosphorylated RTKs through its regulatory subunit p85 and binds to the phosphotyrosine residues. This interaction brings the catalytic subunit p110 within the near vicinity of the plasma membrane, where it can access its lipid substrates. PI3K and phosphorylates phosphatidylinositol (4,5)-bisphosphate (PIP2) is used to generate phosphatidylinositol (3,4,5)-trisphosphate (PIP3). AKT is one of the proteins with pleckstrin homology (PH) domains interact with PIP3 (Glaviano et al., 2023; He et al., 2021). Recruitment to the membrane allows AKT to be phosphorylated and initiated by upstream kinases, including PDK1 (phosphoinositide-dependent kinase-1) and mTORC2 (mechanistic target of rapamycin complex 2). Activated AKT phosphorylates and inhibits the TSC1/TSC2 complex and normally acts as a negative regulator of mTORC1. A key regulator of cell growth and metabolism, mTORC1 is activated as a result of such blockade. mTORC1 phosphorylates subsequent effectors such 4E-BP1 and S6K to stimulate the production of proteins as well as cell proliferation (Panwar et al., 2023; Mishra et al., 2021).

### 2.6 Targeting the PI3K/AKT/mTOR pathway in cancer therapy: Inhibitors and mechanisms

The deregulation of the PI3K/AKT/mTOR pathway in lung cancer is caused by a variety of genetic changes in its constituent parts. These consist of excess or amplified AKT, diminished function variants or deletions in PTEN (a tumour suppressor dephosphorylates PIP3 to PIP2), as well as mutations in PIK3CA (encoding the p110α catalytic subunit of PI3K) and increases oncogenesis (Rascio et al., 2021). Several PI3K inhibitors are in clinical development. These include isoform-specific inhibitors, like Alpelisib (targeting PI3Kα), and pan-PI3K inhibitors like Buparlisib. These inhibitors aim to block the aberrant signaling through the PI3K pathway in cells80. AKT inhibitors, like Capivasertib and Ipatasertib, are designed to prevent subsequent signaling by inhibiting AKT’s kinase function. These inhibitors have shown efficacious results in vivo studies and are undergoing clinical evaluation for their efficacy in lung cancer (Ali et al., 2022). mTOR inhibitors, like Everolimus and Temsirolimus, target the mTORC1 complex, inhibiting its activity and downstream signaling. These drugs have shown efficacy in various cancers, including lung cancer, especially when combined with other therapeutic agents. Resistance to PI3K/AKT/mTOR pathway inhibitors can arise through various mechanisms, including initiation of compensatory pathways (like the MAPK pathway), changes in downstream effectors, and feedback activation of upstream receptors (Son et al., 2024).

### 2.7 ALK pathway

The Anaplastic Lymphoma Kinase (ALK) pathway is involved in cell growth and survival. ALK rearrangements occur in around 3%–7% of cases with NSCLC, most commonly in adenocarcinomas (Son et al., 2024). Patients with ALK-positive lung carcinomas tend to be younger, often non-smokers and have distinct clinical and pathological features compared to other NSCLC subtypes (Grosse et al., 2019). The mechanism of ALK activation in lung cancer is through gene rearrangements. A chromosomal inversion on chromosome 2 causes the echinoderm microtubule-associated protein-like 4 (EML4) gene to merge into the ALK gene, resulting in the EML4-ALK fusion gene. This fusion causes the production of a constitutively active ALK tyrosine kinase drives cancer cell proliferation and survival. The EML4-ALK fusion protein undergoes autophosphorylation, activating its kinase domain (Grosse et al., 2019; Martelli et al., 2009). This activation triggers several downstream signaling pathways critical for tumor growth and survival. These fusions lead to constitutive activation of ALK signaling, promoting tumorigenesis (Shreenivas et al., 2023; Holla et al., 2017). The first ALK inhibitor was approved for ALK-positive NSCLC. Crizotinib inhibits ALK, ROS1, and MET kinases, leading to tumor regression and improved progression-free survival. However, resistance to Crizotinib often develops within a year of treatment initiation. Designed to overcome Crizotinib resistance, Ceritinib has shown efficacy in patients with Crizotinib-resistant ALK-positive NSCLC (Ou et al., 2012; Shaw et al., 2020). Another second-generation inhibitor with improved efficacy and central nervous system (CNS) penetration, Alectinib is effective against Crizotinib-resistant and brain metastases in ALK-positive NSCLC. Brigatinib (Alunbrig): Exhibits activity against various ALK resistance mutations and has shown significant efficacy in both Crizotinib-resistant and treatment-naïve ALK-positive NSCLC patients. Third-generation ALK inhibitors like Lorlatinib (Lorbrena) are intended to suppress tolerance to their predecessors. Lorlatinib has shown activity against a broad range of ALK resistance mutations and has good CNS penetration (Delmonte et al., 2019; Lin et al., 2018; Yang et al., 2020).

### 2.8 MET pathway

The MET pathway, activated by the Hepatocyte Growth Factor (HGF), regulates cell growth, survival, and metastasis. When the TK receptor MET binds to its ligand, HGF, sometimes referred to as the scatter factor, it becomes active (Organ and Tsao, 2011). MET receptor dimerization and autophosphorylation are triggered by HGF binding at certain tyrosine residues in the intracellular domain. Phosphorylated tyrosine residues on MET act as docking points for multiple enzyme and adaptor protein molecules and starts several signaling cascades downstream. The genetic alterations prevent the degradation of the MET receptor, leading to its prolonged activation (Uchikawa et al., 2021; Zhang et al., 2018). Roughly 3%–4% of NSCLC patients had MET exon 14 skipping mutations. Overexpression of the MET receptor and increased signaling result from an increase in MET gene copies. In around 2%–5% of NSCLC patients, MET amplification is seen. Increased expression of the MET receptor or its ligand HGF can drive oncogenic signaling in lung cancer cells (Socinski et al., 2021; Heist et al., 2016). These consist of monoclonal antibodies as well as small-molecule TKIs. Initially approved for ALK-positive NSCLC, Crizotinib also inhibits MET and has shown efficacy in patients with MET exon 14 skipping mutations or MET amplification (Feng and Ma, 2012; Spagnolo et al., 2023). Specifically designed to inhibit MET, Capmatinib is approved for the treatment of NSCLC with MET exon 14 skipping mutations. It has shown significant clinical activity in both treatment-naïve and previously treated patients. Another MET-specific inhibitor, Tepotinib is approved for NSCLC with MET exon 14 skipping mutations, demonstrating durable responses in clinical trials. In MET-positive NSCLC, onartuzumab, a monoclonal antibody targets the MET receptor, was recently investigated in conjunction with EGFR inhibitors. However, clinical trials have shown mixed results, and its development has been limited (Wolf et al., 2024; Jørgensen et al., 2024; Wolf et al., 2020).

### 2.9 Notch pathway

The division and viability of cells are impacted by the Notch signaling system. Lung cancer has been linked to Notch signaling instability. Greater tumor cell proliferation as well as resistance to apoptosis can result from aberrant Notch activation. The Notch pathway starts when a ligand from the Jagged or Delta-like (DLL) families is present on a neighboring cell attaches itself to a Notch receptor on the cell surface. There are four Notch receptors (Notch 1–4) and several ligands (DLL3, DLL1, DLL4, Jagged2, Jagged1) (Capaccione et al., 2013; Yuan et al., 2014). The Notch receptor undergoes a conformational change upon ligand interaction, exposing a cleavage site for the ADAM (a Disintegrin and metalloprotease) class of proteases. The Notch intracellular domain (NICD) is then released when the receptor is first cleaved by ADAM proteases and then again by the γ-secretase unit. Recombination signal binding protein for immunoglobulin kappa J region, or CSL, is a DNA-binding protein the NICD connects with after translocating to its nucleus. This complex recruits’ coactivators, leading to the transcription of Notch target genes, like Hey and Hes family members and are involved in cell fate decisions. Dysregulation of the Notch pathway can occur through overexpression of Notch receptors or ligands, loss of negative regulators, or mutations lead to constitutive activation of pathway (Zhou et al., 2022; Wang et al., 2011). Loss-of-function mutations in Notch receptors, particularly Notch1 and Notch2, have been identified, leading to uncontrolled cell growth and tumorigenesis. γ-Secretase Inhibitors (GSIs) block the cleavage of Notch receptors by the γ-secretase complex, preventing the release of the NICD and subsequent transcriptional activation of target genes (Chen et al., 2025). GSIs like RO4929097 and MK-0752 have been investigated in clinical studies to see how well they address Notch-dependent cancers (Czerwonka et al., 2023). However, their se is limited by significant side effects, including gastrointestinal toxicity. Monoclonal antibodies targeting Notch receptors or ligands can inhibit Notch signaling. For example, demcizumab (anti-DLL4 antibody) has being investigated for its capacity to prevent lung cancer tumour development and angiogenesis (You et al., 2023; Xia et al., 2022).

### 2.10 Wnt/β-catenin pathway

**FIGURE 5:**
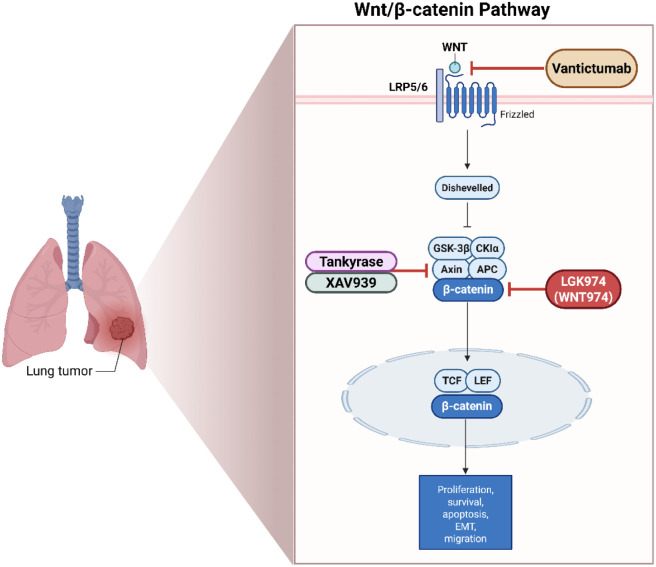
Illustrates the Wnt/β-catenin signaling pathway and its targeted inhibition in lung cancer. Binding of Wnt ligands to the Frizzled receptor and LRP5/6 co-receptor activates the pathway by stabilizing β-catenin through inhibition of its degradation complex (comprising GSK-3β, CKIα, Axin, and APC). Stabilized β-catenin translocates to the nucleus, where it interacts with TCF (T-cell factor) and LEF (lymphoid enhancer-binding factor) transcription factors to regulate genes controlling proliferation, survival, apoptosis, epithelial–mesenchymal transition (EMT), and migration. Therapeutic inhibitors block various steps of the pathway: Vantictumab inhibits Wnt ligand-receptor binding; LGK974 (WNT974) targets β-catenin; and XAV939 inhibits Tankyrase, stabilizing Axin and promoting β-catenin degradation. These inhibitors represent promising strategies to suppress aberrant Wnt signaling in lung tumors. Created in https://BioRender.com Abbreviations: APC, adenomatous polyposis coli; EMT, epithelial-to-mesenchymal transition; GSK-3β, glycogen synthase kinase 3 beta; LEF, lymphoid enhancer-binding factor; TCF, T-cell factor.

The Wnt/β-catenin pathway processes cell division, migration, as well as differentiation. When Wnt ligands attach to a cell surface receptor complex made up of Frizzled (FZD) receptors and LRP5/6 co-receptors, the Wnt/β-catenin pathway is triggered (Figure 5) (Xue et al., 2025; Hu et al., 2024). As illustrated in Figure 5, Wnt/β-catenin signaling stabilizes β-catenin to drive proliferation and EMT in lung tumors, revealing opportunities for inhibitors like LGK974 to disrupt this oncogenic cascade. This binding induces the formation of a signalosome, inhibiting β-catenin. When Wnt signaling is not present, β-catenin is constantly broken down by a destruction complex made up of the subunits CK1 (casein kinase 1), Axin, GSK-3β (glycogen synthase kinase 3 beta), and APC (adenomatous polyposis coli) (Gao et al., 2014). Wnt ligand binding inhibits this complex, stopping the breakdown of β-catenin. After building up in the cytoplasm, stabilized β-catenin moves into the nucleus and attaches itself to the transcription factors TCF/LEF (T-cell factor/lymphoid enhancer factor). The transcription of Wnt target genes regulate cell motility, survival, and reproduction is activated by this process (Qin et al., 2024; Stamos and Weis, 2013).

### 2.11 Wnt/β-catenin signaling pathway and therapeutic targets in lung cancer

Abnormal activity of the Wnt/β-catenin pathway in lung cancer can occur through various mechanisms, including overexpression of Wnt ligands or receptors, mutations in β-catenin or APC, and loss of negative regulators. These alterations cause the route to be activated abnormally, promoting tumorigenesis (Liu et al., 2022). The formation and progression of lung cancer are facilitated by inappropriate activation of the Wnt/β-catenin pathway and increases cell entry, longevity, and replication (Song et al., 2024; Zhang Z. et al., 2024). β-catenin can interact with other signaling pathways, including EGFR and PI3K/AKT and may further contribute to tumor development and treatment resistance (Zhu et al., 2022). Porcupine is an enzyme required for Wnt ligand secretion120. Inhibitors like LGK974 (WNT974) block Wnt secretion, reducing Wnt signaling and tumor growth (Porcupine inhibitor LGK-974 inhibits Wnt/β-catenin signaling and modifies tumor-associated macrophages resulting in inhibition of the malignant behaviors of non-small cell lung cancer cells) (Liu et al., 2013). Tankyrase is engaged in the control of Axin, a component of the β-catenin destruction complex. Inhibitors like XAV939 stabilize Axin, promoting β-catenin degradation (Huang et al., 2009). Monoclonal antibodies targeting Wnt ligands or receptors can inhibit Wnt signaling (Yu et al., 2021). For example, OMP-18R5 (Vantictumab) targets multiple Frizzled receptors, blocking Wnt ligand binding and signaling (Diamond et al., 2020).

### 2.12 Kinases as therapeutic targets

Targeting kinases has become a pivotal strategy in cancer therapy, particularly in lung cancer, due to their central roles in signalling pathways control the ability to survive and proliferation of cells, and metastasis (Fatma et al., 2025). Kinases like EGFR, c-MET (Mesenchymal-Epithelial Transition Factor), Anaplastic Lymphoma Kinase (ALK), BRAF, and ROS1 are frequently implicated in lung cancer. Each of these kinases can harbor mutations or undergo alterations lead to their continuous activation, promoting uncontrolled cell growth and survival. Targeting these aberrant kinases with specific inhibitors has significantly advanced the treatment of lung cancer (Jiang et al., 2025; Chevallier et al., 2021; Chan and Hughes, 2015).

### 2.13 Role of kinases in cancer cell proliferation and survival

EGFR is one of the most well-studied kinases in lung cancer. Tumor development is fueled by mutations in the EGFR gene, like L858R and exon 19 deletions and continuously activate downstream signaling pathways such PI3K/AKT and RAS/RAF/MEK/ERK. EGFR TKIs like erlotinib, gefitinib, afatinib, and Osimertinib have been created to block this activity, showing remarkable efficacy in patients with these specific mutations. Similarly, ALK rearrangements, like the EML4-ALK fusion, result in constitutive kinase activity. ALK inhibitors like crizotinib, ceritinib, and alectinib effectively target these alterations, providing significant clinical benefits (Degirmenci et al., 2020; Hoang et al., 2020)^.^

c-MET, another critical kinase, is triggered by hepatocyte growth factor (HGF). Mutations or overexpression of c-MET can enhance cancer cell proliferation, survival, invasion, and metastasis. Inhibitors like crizotinib, capmatinib, and tepotinib block c-MET signaling, showing effectiveness, particularly in tumors with MET exon 14 skipping mutations. BRAF mutations, notably V600E, activate the MAPK/ERK pathway, promoting lung cancer cell proliferation. BRAF inhibitors like dabrafenib and vemurafenib target this kinase, providing another tailored treatment option. ROS1 rearrangements, found in a portion of lung cancer, are targeted by inhibitors like crizotinib and entrectinib, further expanding the arsenal against lung cancer (Feng and Ma, 2012; Śmiech et al., 2020; Santarpia et al., 2021). The mechanisms of kinase inhibitors are diverse. Most are ATP-competitive, attaching to the kinase’s ATP-binding site to prevent phosphorylation of substrates. Others are allosteric inhibitors, binding to different sites to induce conformational changes inactivate the kinase. Some inhibitors form covalent bonds with the kinase, leading to prolonged inhibition (Thomson et al., 2023).

## 3 Challenges in targeting kinases

### 3.1 Resistance mechanisms

Resistance to kinase inhibitors in therapy is a significant clinical challenge, hindering the long-term effectiveness of these treatments. Resistance can be classified into primary resistance, where patients exhibit no initial response to the therapy, and acquired resistance, where tumors initially respond but later progress despite continued treatment. The emergence of additional mutations inside the kinase domain constitutes one of the key resistance mechanisms. Mutations like these can decrease the inhibitor’s affinity for binding by changing the structure of the ATP-binding pocket or other significant components of the kinase (Barouch-Bentov and Sauer, 2011). For instance, in NSCLC treated with EGFR inhibitors like gefitinib or erlotinib, a common secondary mutation, T790M, emerges. This mutation increases the affinity of the ATP binding site for ATP and diminishing the efficacy of the ATP-competitive inhibitors. To address this, the T790M mutation is the specific target of third-generation EGFR inhibitors like Osimertinib (Zhang, 2016).

Alternate signaling pathways might avoid the inhibited kinase are activated as another resistance strategy, maintaining the growth and survival of cells. Tumor cells can activate compensatory pathways through mutations or amplifications in other genes. For example, when EGFR inhibitor resistance occurs, amplification of the MET gene can activate the MET/HGF pathway and can substitute for EGFR signaling. This activation causes continued downstream signaling through the PI3K/AKT and RAS/RAF/MEK/ERK pathways, rendering the EGFR inhibition ineffective. In such cases, combining EGFR inhibitors with MET inhibitors may provide a therapeutic benefit by simultaneously blocking both pathways (Ko et al., 2017).

Phenotypic changes in tumor cells also contribute to resistance. One well-documented change is the epithelial-to-mesenchymal transition (EMT), a mechanism by which cancer cells get a more invasive and motile phenotype. EMT is associated with a loss of epithelial markers and an increase in mesenchymal markers and can alter the dependency of cancer cells on specific signaling pathways. For example, during EMT, cells may become less reliant on EGFR signaling and more dependent on other survival pathways, thus diminishing the efficacy of EGFR inhibitors. This transition not only confers resistance but also enhances the metastatic potential of cancer cells, complicating treatment further (Nurwidya et al., 2012).

Gene amplification is an additional way cancer cells become resistant. Amplification of the target kinase itself or other kinases within the same signaling network can overwhelm the inhibitor’s ability to effectively block the pathway. For instance, amplification of the HER2 gene may cause resistance to EGFR inhibitors, as the increased HER2 signaling can compensate for the inhibited EGFR pathway. Targeting both EGFR and HER2 simultaneously has being investigated as a means of overcoming this opposition (Gan et al., 2021).

Tumor microenvironment situations also cause resistance. Interactions between cancerous cells and their surrounding immune cells, stroma, and extracellular matrix can influence drug response. For example, cancer-associated fibroblasts (CAFs) can cytokines and secrete growth factors activate alternative survival pathways in tumor cells and promoting resistance to targeted therapies. hypoxic conditions within the tumor microenvironment can induce adaptive responses enhance cell survival and resistance to treatment (Kansal and Agrawal, 2025; Fiori et al., 2019).

### 3.2 Side effects and off-target effects

**FIGURE 6:**
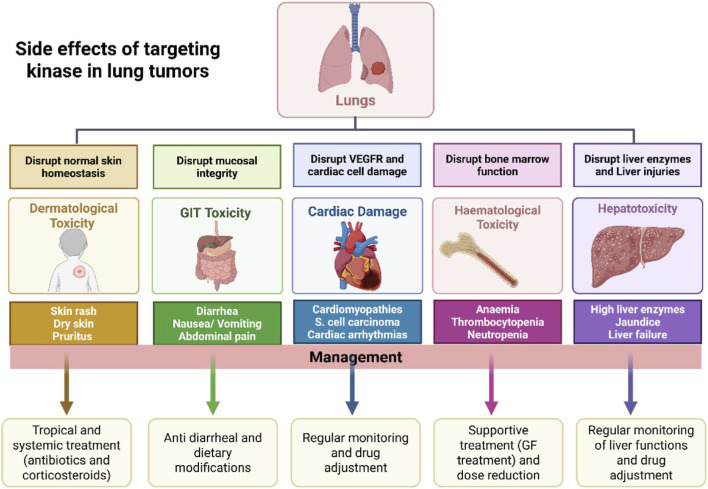
Presents the common side effects associated with targeting kinases in lung tumor therapy and outlines their respective management strategies. Kinase inhibitors, while effective, may disrupt various physiological systems, leading to toxicities. Dermatological toxicity results from disrupted skin homeostasis and includes symptoms such as rash, dry skin, and pruritus, managed with topical/systemic antibiotics or corticosteroids. Gastrointestinal (GIT) toxicity, caused by mucosal integrity disruption, presents as diarrhea, nausea, vomiting, and abdominal pain, managed with antidiarrheal agents and dietary changes. Cardiac damage may arise from VEGFR disruption, leading to cardiomyopathies and arrhythmias, necessitating regular monitoring and dose adjustments. Hematological toxicity, due to bone marrow suppression, causes anemia, thrombocytopenia, and neutropenia, managed with supportive therapies like growth factors and dose modifications. Hepatotoxicity, resulting from impaired liver function, manifests as elevated liver enzymes, jaundice, or liver failure, requiring regular liver function monitoring and drug dose adjustment. Created in https://BioRender.com Abbreviations: VEGFR, vascular endothelial growth factor receptor.

A significant challenge associated with these drugs is the occurrence of side effects and off-target effects. These adverse reactions arise because, despite their targeted nature, kinase inhibitors often affect multiple kinases due to the structural similarities in their ATP-binding sites. This lack of absolute specificity can lead to unintended inhibition of kinases vital for normal cellular functions, resulting in a range of side effects (Figure 6) (Shyam Sunder et al., 2023). As illustrated in Figure 6, kinase inhibitors induce dermatological, gastrointestinal, and cardiac toxicities via off-target effects, illustrating the need for selective agents and monitoring strategies.

Dermatological toxicity is among kinase inhibitors’ most frequent adverse effects. For example, skin rashes, dry skin, and pruritus are common side effects seen by EGFR inhibitor-treated individuals. The underlying mechanism is linked to the inhibition of EGFR in the skin and is crucial for normal skin homeostasis and repair. The skin rash, often presenting as acneiform eruptions, not only affects patients’ quality of life but can also lead to dose modifications or discontinuation of the therapy. Management strategies for these dermatological side effects include topical and systemic treatments, like antibiotics and corticosteroids, but these interventions do not always completely alleviate the symptoms (Li et al., 2022a; Holcmann and Sibilia, 2015).

Gastrointestinal toxicity is another prevalent side effect associated with kinase inhibitors. Drugs targeting EGFR, ALK, and other kinases often cause diarrhea, nausea, vomiting, and abdominal pain. These symptoms are brought on by the gastrointestinal tract’s kinase activity being inhibited and disrupts normal cellular turnover and mucosal integrity. For example, EGFR is expressed in the epithelial cells of the gut, and its inhibition can lead to impaired absorption and increased secretion, contributing to diarrhea. Effective management of gastrointestinal toxicity includes supportive care measures like anti-diarrheal agents and dietary modifications, but severe cases may necessitate dose reductions or treatment interruptions (Tao and Chityala, 2021).

One serious and sometimes fatal adverse effect of certain kinase inhibitors is cardiovascular damage. For instance, BRAF inhibitors, used to treat melanoma and other malignancies, have been linked to the growth of cardiomyopathies and cutaneous squamous cell carcinomas. Drugs like crizotinib, an ALK inhibitor, can cause QT prolongation, leading to an increased risk of cardiac arrhythmias. The cardiotoxic effects are attributed to the off-target inhibition of kinases involved in cardiac function, like the vascular endothelial growth factor receptor (VEGFR). Regular monitoring of cardiac function and appropriate dose adjustments are crucial in managing these risks (Pun and Neilan, 2016).

Hepatotoxicity is another significant concern with kinase inhibitors. Many of these drugs are metabolized in the liver, and their accumulation can lead to liver injury. Elevated liver enzymes, jaundice, and in severe cases, liver failure, have been reported with various kinase inhibitors. The mechanisms involve direct hepatocyte toxicity and immune-mediated liver damage. Monitoring liver function tests regularly and adjusting doses as necessary are essential steps in managing hepatotoxicity (Viganò et al., 2023).

### 3.3 Side effects and management of kinase inhibitor therapy in lung tumors

Off-target effects also extend to hematological toxicity, where kinase inhibitors can disrupt normal bone marrow function, leading to anemia, thrombocytopenia, and neutropenia (Gharwan and Groninger, 2016). For example, multitargeted kinase inhibitors like sunitinib, used in renal cell carcinoma and gastrointestinal stromal tumors, can cause significant myelosuppression. This toxicity results from the inhibition of kinases play roles in hematopoiesis, like the stem cell factor receptor (KIT). Managing hematological toxicity often requires supportive treatments like growth factors or transfusions and may necessitate dose reductions (Kollmannsberger et al., 2011).

The challenge of off-target effects underscores the need for developing more selective kinase inhibitors. To increase specificity, one strategy is to create allosteric inhibitors attach to locations different from the ATP-binding region (Li J. et al., 2024). Another approach is developing covalent inhibitors irreversibly bind to unique cysteine residues in the target kinase, reducing off-target interactions (Huang et al., 2022). combination therapies use lower doses of multiple drugs targeting different pathways may mitigate the side effects while maintaining therapeutic efficacy (Fan et al., 2025).

## 4 Natural products as kinase inhibitors

**FIGURE 7:**
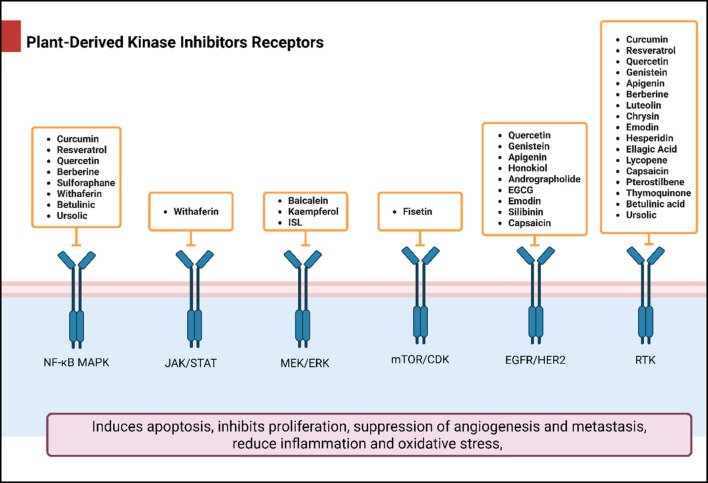
Illustrates various plant-derived bioactive compounds that act as natural kinase inhibitors, targeting key signaling receptors and pathways implicated in lung cancer development and progression. These pathways include NF-κB/MAPK, JAK/STAT, MEK/ERK, mTOR/CDK, EGFR/HER2, and RTKs (Receptor Tyrosine Kinases). Compounds such as Curcumin, Resveratrol, Quercetin, Berberine, Withaferin, Genistein, Apigenin, Capsaicin, and many others inhibit specific kinases, leading to multiple anticancer effects. These include induction of apoptosis, inhibition of cell proliferation, suppression of angiogenesis and metastasis, and reduction of inflammation and oxidative stress. Their multitargeted action positions them as promising complementary agents in cancer therapy, particularly for overcoming resistance to conventional kinase inhibitors. Created in https://BioRender.com Abbreviations: EGFR, epidermal growth factor receptor; ERK, extracellular signal-regulated kinase; HER2, human epidermal growth factor receptor 2; JAK, Janus kinase; MAPK, mitogen-activated protein kinase; MEK, mitogen-activated protein kinase; STAT, signal transducer and activator of transcription; mTOR, mechanistic target of rapamycin.

Natural compounds refer to bioactive substances obtained from organic sources like plants, marine organisms, and microorganisms. These compounds have been utilized in traditional therapy for generations and have garnered significant interest in modern biomedical research due to their diverse therapeutic properties in cell signaling pathways (Wang et al., 2025; Kong et al., 2025) (Figure 7). As illustrated in Figure 7, plant-derived NKIs like curcumin and resveratrol multitarget pathways such as EGFR/HER2 and MEK/ERK, positioning them as low-toxicity adjuncts to overcome resistance.

Beyond their well-established molecular and preclinical activity, natural kinase inhibitors (NKIs) are increasingly being explored for their clinical relevance in lung cancer management. Several plant-derived compounds such as curcumin, resveratrol, quercetin, genistein, and epigallocatechin gallate (EGCG) have progressed from *in vitro* and *in vivo* studies toward early-phase clinical evaluation, primarily as adjuncts to conventional therapies. These agents modulate clinically relevant kinase-driven pathways including EGFR, PI3K/AKT/mTOR, MAPK, and JAK/STAT, which are frequently dysregulated in non-small cell lung cancer (NSCLC). Their multitargeted mechanism, favorable safety profile, and low systemic toxicity make them attractive candidates for long-term use and combination regimens in clinical settings.

Several natural compounds, including curcumin, resveratrol, quercetin, genistein, and epigallocatechin gallate (EGCG), have demonstrated clinically relevant kinase modulation in human studies and early-phase trials. These agents target key oncogenic pathways such as EGFR, PI3K/AKT/mTOR, MAPK, and JAK/STAT, which are central to lung cancer progression.

In this review, clinical evidence is primarily discussed in the context of natural kinase inhibitors and their role in combination-based therapeutic strategies, rather than as standalone replacements for approved synthetic drugs. Natural compounds such as curcumin, resveratrol, quercetin, genistein, and epigallocatechin gallate (EGCG) have demonstrated clinically relevant modulation of kinase-driven pathways, particularly when used in conjunction with chemotherapy or targeted therapies. Emerging clinical and translational studies suggest that these agents may enhance therapeutic sensitivity, mitigate drug-induced toxicity, and modulate resistance-associated signaling pathways in lung cancer.

Unlike synthetic kinase inhibitors that are primarily evaluated as monotherapies, natural kinase inhibitors are increasingly investigated for their role as adjunct therapeutic agents in lung cancer management. Early-phase clinical studies indicate that natural compounds such as curcumin and resveratrol are well tolerated and may enhance the efficacy of chemotherapy and targeted therapies by modulating kinase-driven resistance mechanisms, reducing inflammation, and improving cellular sensitivity to treatment. Importantly, these compounds exhibit low systemic toxicity, supporting their potential utility in long-term or combination-based clinical strategies.

### 4.1 Plant-derived kinase inhibitors and their target receptors in cancer therapy in non-small cell lung cancer (NSCLC)

### 4.2 Advantages of natural compounds

Natural compounds generally exhibit fewer side effects compared to synthetic drugs (Tessema et al., 2024). This is partly because they have co-evolved with biological systems and are often better tolerated by the human body. Many natural compounds have the ability to target multiple signaling pathways simultaneously, providing a broad-spectrum approach to cancer therapy. Grape resveratrol alongside turmeric’s curcumin have shown anticancer properties with minimal toxicity in clinical studies (Jain et al., 2024a; Jain et al., 2023a; Jain et al., 2023b). Compounds like quercetin and Epigallocatechin Gallate (EGCG) (from green tea) are known to inhibit various kinases and other molecules involved in cancer progression (Kciuk et al., 2023). Natural compounds can enhance the effectiveness of conventional treatments like radiation or chemotherapy. By acting synergistically, these compounds can increase the effectiveness of traditional therapies while potentially reducing their required doses and associated side effects. Curcumin has been shown to improve the reaction to treatment in a number of cancers forms (Mehta et al., 2014).

Numerous natural substances have anti-inflammatory as well as antioxidant qualities and may help explain their antitumor benefits. These properties help in reducing oxidative stress and inflammation, both of have been linked to the growth of cancer and its progression (Imran et al., 2019; Zheng et al., 2024). Resveratrol and genistein (from soy) are examples of compounds with strong antioxidant and anti-inflammatory activities (Banerjee et al., 2008). The use of natural compounds may lower the risk of developing drug resistance (Singh et al., 2020), because they often target multiple pathways and cellular processes, cancer cells may find it more challenging to adapt and develop resistance compared to single-target synthetic drugs. This makes natural compounds promising candidates for long-term cancer management. Natural compounds are often more accessible and cost-effective compared to synthetic drugs. Many of these compounds can be sourced from readily available plants and foods, making them an affordable option for cancer prevention and treatment, particularly in resource-limited settings. Given their relatively low toxicity, natural compounds can be used not only for treatment but also for cancer prevention. Regular consumption of foods rich in natural bioactive compounds, like fruits, vegetables, and teas, can contribute to reducing the risk of cancer development (Mali, 2023).

From a clinical standpoint, natural kinase inhibitors offer several advantages over conventional synthetic kinase inhibitors. Early-phase clinical studies and human supplementation trials have demonstrated that compounds such as curcumin and resveratrol are generally well tolerated, even at relatively high doses, with minimal adverse effects. Importantly, these compounds have been reported to enhance the therapeutic response to EGFR tyrosine kinase inhibitors and chemotherapy by sensitizing tumor cells, reducing inflammation-mediated resistance, and modulating survival pathways. Although their clinical efficacy as monotherapies remains limited due to bioavailability challenges, NKIs show promise as supportive or adjunct therapeutic agents capable of improving treatment tolerability and potentially delaying resistance development.

### 4.3 Natural sources of kinase inhibitors

#### 4.3.1 Plant-derived kinase inhibitors

Plant-derived kinase inhibitors are bioactive compounds obtained from various plant sources exhibit the ability to inhibit kinase activity. These organic substances have demonstrated encouraging promise in cancer treatment by focusing on important signaling pathways.

Clinical evidence supporting plant-derived kinase inhibitors in lung cancer is emerging but remains at an early stage. Curcumin has been evaluated in multiple Phase I and II clinical trials in patients with solid tumors, including lung cancer, demonstrating safety and potential improvement in treatment-related outcomes when used alongside chemotherapy or targeted therapy. Resveratrol and quercetin have shown favorable pharmacodynamic effects in human studies by modulating PI3K/AKT and MAPK signaling, although their direct clinical efficacy in lung cancer requires further validation. EGCG has been investigated in clinical settings for its anti-proliferative and anti-angiogenic effects, with evidence supporting its role in modulating EGFR-related signaling. Collectively, these findings suggest that while most natural kinase inhibitors have not yet achieved standalone clinical approval for lung cancer, they possess translational potential, particularly in combination-based therapeutic strategies.

Clinical evaluation of plant-derived kinase inhibitors remains at an early stage but provides encouraging translational insights. Curcumin has been assessed in Phase I and II clinical trials involving patients with solid tumors, including lung cancer, demonstrating safety and potential improvement in treatment response when used as an adjunct to conventional therapies. Resveratrol and quercetin have shown favorable pharmacodynamic effects in human studies by suppressing PI3K/AKT and MAPK signaling pathways. EGCG has been reported to influence EGFR-related signaling in clinical and translational studies. While these natural compounds have not yet achieved standalone regulatory approval for lung cancer, available human evidence supports their adjunctive clinical potential, justifying continued investigation.

### 4.4 Marine-derived kinase inhibitors

Marine-derived kinase inhibitors are bioactive compounds obtained from various marine organisms, including sponges, algae, bryozoans, and microorganisms (Table 1).

**TABLE 1:** Bioactive compounds from marine organisms offer promising therapeutic potential for drug discovery and disease treatment.

Compound	Chemical Structure	Source	Kinases Inhibited	Mechanisms of Action	Anti-Cancer Effects	References
Bryostatin	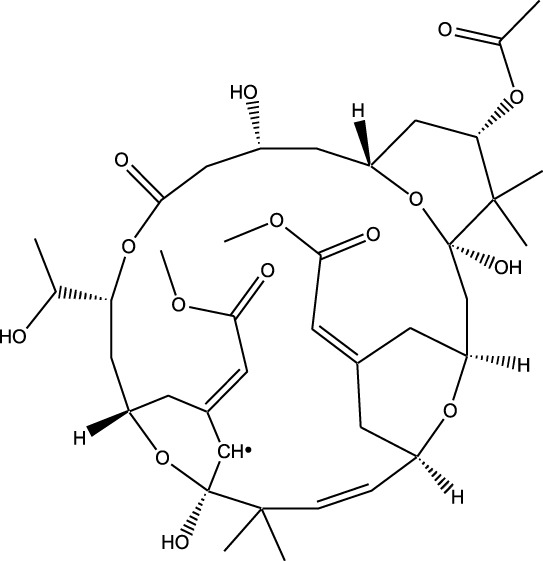	Marine bryozoans (*Bugula neritina*)	PKC	Inhibits PKC, modulates immune response	Induces apoptosis, enhances efficacy of other anti-cancer agents	Ly et al. (2020)
Discodermolide	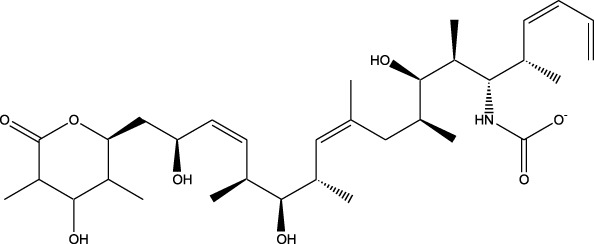	Marine sponges (*Discodermia dissoluta*)	Microtubules (indirect kinase inhibition)	Stabilizes microtubules, affects kinases involved in cell cycle regulation	Induces apoptosis, effective in drug-resistant cancer cell lines	Ly et al. (2020)
Dolastatin 10	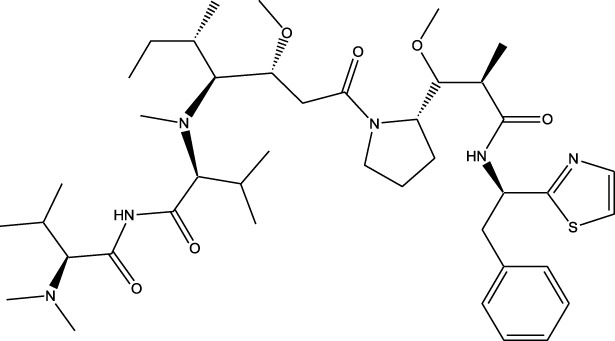	Marine sea hare (*Dolabella auricularia*)	Microtubules (indirect kinase inhibition)	Disrupts microtubule dynamics, affects cell cycle regulation kinases	Induces apoptosis, significant anti-cancer activity	Gutman et al. (2021)
Peloruside A	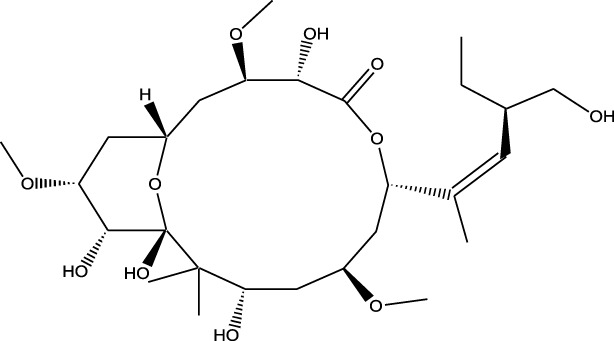	Marine sponge (*Mycale hentscheli*)	Microtubules (indirect kinase inhibition)	Binds to microtubules, stabilizes microtubule dynamics	Induces apoptosis, inhibits proliferation	Risinger and Du (2020)

Abbreviations: PKC, Protein Kinase C.

### 4.5 Microbial-derived kinase inhibitors

Microbial-derived kinase inhibitors are bioactive compounds obtained from various microorganisms, including bacteria, fungi, and actinomycetes (Table 2).

**TABLE 2:** Microbial-derived kinase inhibitors offer promising therapeutic potential in targeted cancer treatment and drug discovery.

Compound	Chemical Structure	Source	Kinases Inhibited	Mechanisms of Action	Anti-Cancer Effects	References
Staurosporine	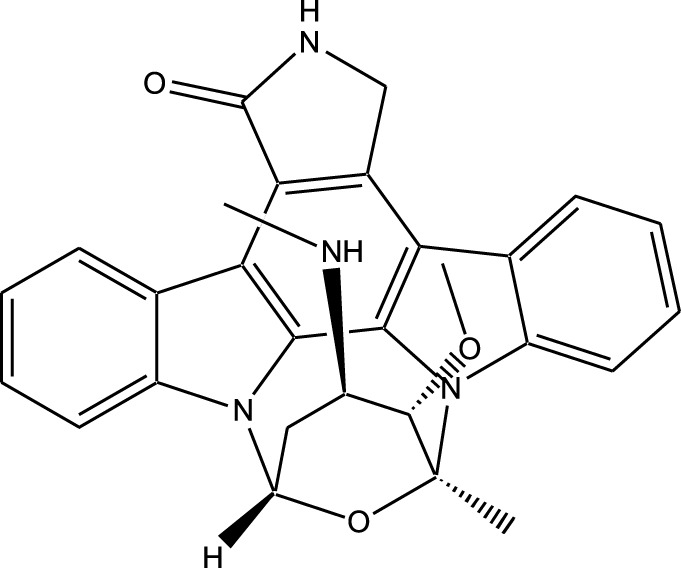	Bacterium (*Streptomyces sp*.)	PKC, CDKs, PKA	Inhibits multiple kinases, including PKC and CDKs	Induces apoptosis, inhibits cell proliferation, foundational for synthetic derivatives like Midostaurin	Tarvainen (2021)
Rapamycin	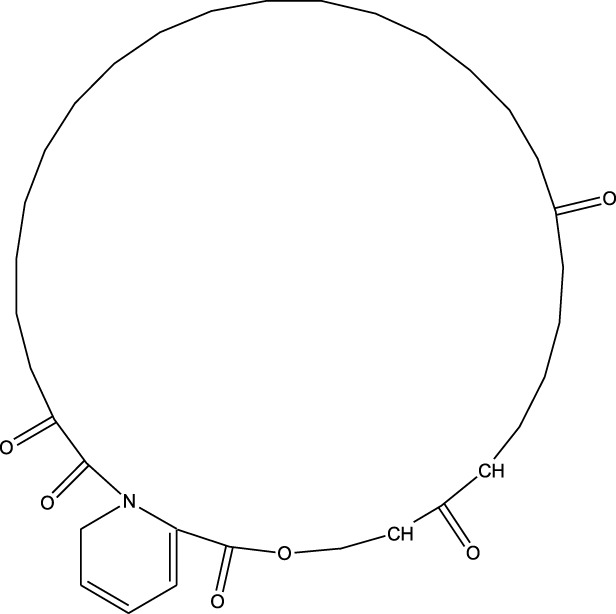	Bacterium (*Streptomyces hygroscopicus*)	mTOR	Inhibits mTOR signalling pathway	Induces apoptosis, inhibits cell proliferation	Querfurth and Lee (2021)
Epothilone B	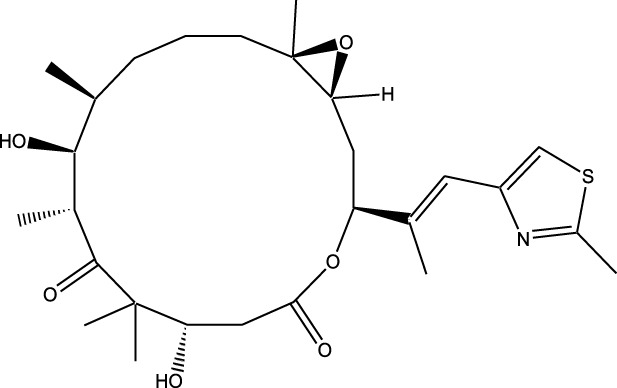	Bacterium (*Sorangium cellulosum*)	Microtubules (indirect kinase inhibition)	Binds to microtubules, stabilizes microtubule dynamics	Induces apoptosis, inhibits cell proliferation	Chen et al. (2020)
Geldanamycin	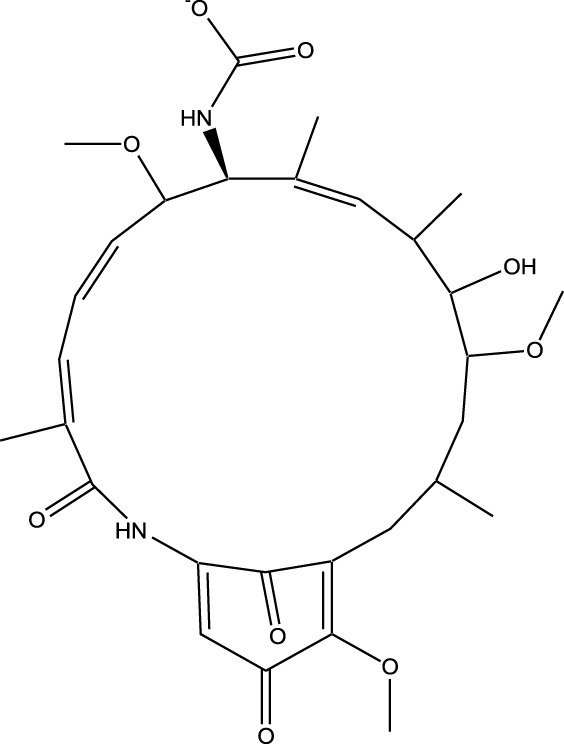	Bacterium (*Streptomyces hygroscopicus*)	Hsp90 (indirect kinase effects)	Inhibits Hsp90, leading to degradation of client proteins including kinases	Induces apoptosis, inhibits cell proliferation	Liew et al. (2022)
Tunicamycin	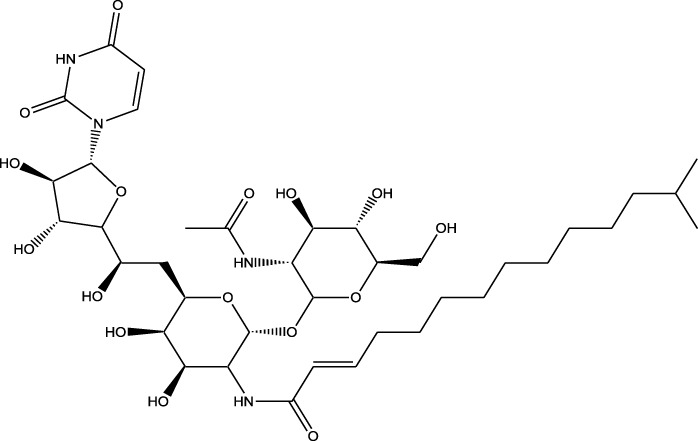	Bacterium (*Streptomyces* sp.)	N-linked glycosylation (indirect kinase effects)	Inhibits protein glycosylation, affecting kinase receptor signaling	Induces apoptosis, inhibits cell proliferation	Dawood and Altobje (2020)
Rocaglamide	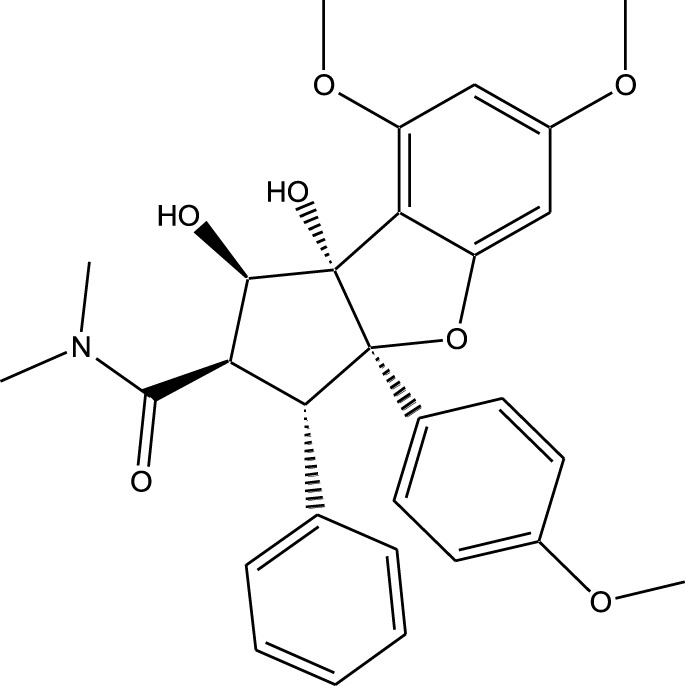	Fungus (*Aglaia species*)	eIF4A (indirect kinase effects)	Inhibits eIF4A, affecting translation initiation and cell cycle regulation	Induces apoptosis, inhibits cell proliferation	Oblinger et al. (2024)

### 4.6 Synthetic and semi-synthetic derivatives

Synthetic and semi-synthetic derivatives of NKIs are chemically modified versions of naturally occurring compounds. These modifications aim to enhance the bioavailability, potency, stability, and selectivity of the original molecules, making them more effective as therapeutic agents (Table 3).

**TABLE 3:** Table showcasing synthetic and semi-synthetic kinase inhibitors and their therapeutic potential in targeted treatments.

Derivative	Parent Compound	Chemical Structure	Source of Parent Compound	Kinases Inhibited	Mechanisms of Action	Anti-Cancer Effects	References
Midostaurin	Staurosporine	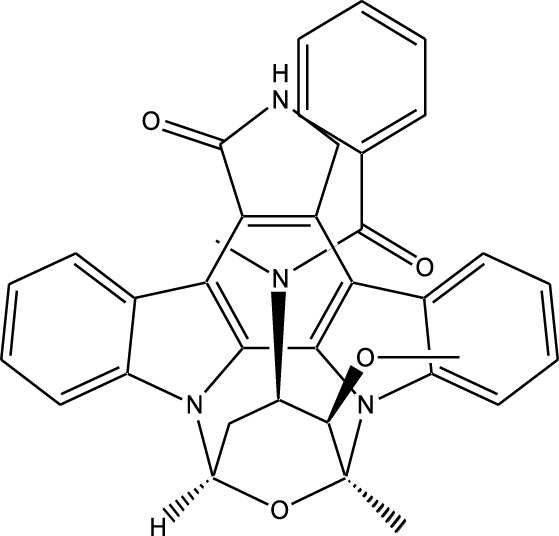	Bacterium (*Streptomyces sp*.)	FLT3, PKC, c-Kit	Inhibits FLT3 and other kinases, used in combination with another chemotherapeutics	Induces apoptosis, inhibits cell proliferation	Gorecki et al. (2025)
Erlotinib	Quercetin	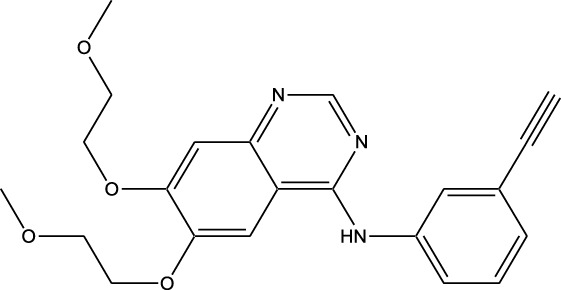	Various plants (fruits, vegetables)	EGFR	Inhibits EGFR tyrosine kinase	Induces apoptosis, inhibits cell proliferation	Ganthala et al. (2022)
Gefitinib	Quercetin	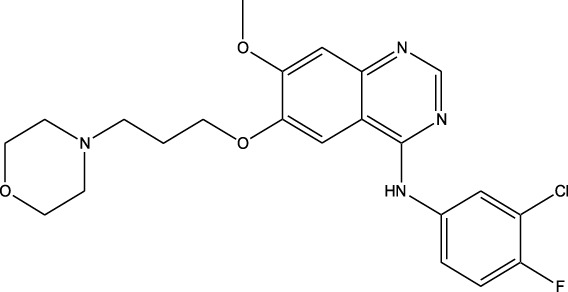	Various plants (fruits, vegetables)	EGFR	Inhibits EGFR tyrosine kinase	Induces apoptosis, inhibits cell proliferation, used	Ge et al. (2023)
Bortezomib (Velcade)	Lactacystin	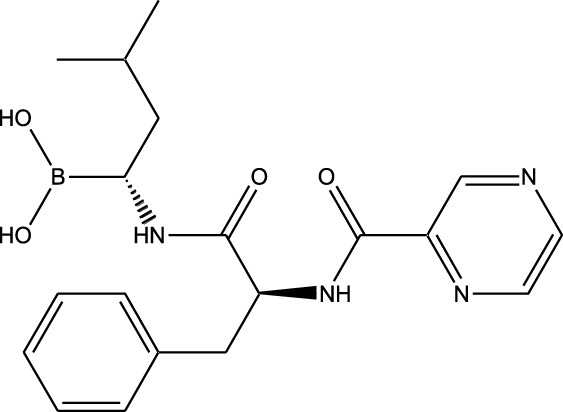	Bacterium (*Streptomyces sp*.)	Proteasome	Inhibits proteasome, affecting protein degradation and cell cycle regulation	Induces apoptosis, inhibits cell proliferation	Fricker (2020)
Lenvatinib (Lenvima)	Resveratrol	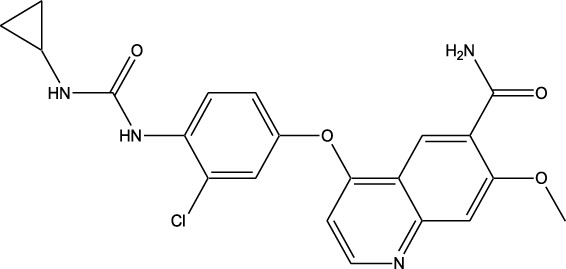	Grapes, berries, peanuts, red wine	VEGFR, FGFR, PDGFR	Inhibits multiple receptor tyrosine kinases	Inhibits cell proliferation, reduces angiogenesis	Singh et al. (2023)
Cabozantinib (Cometriq)	Quercetin	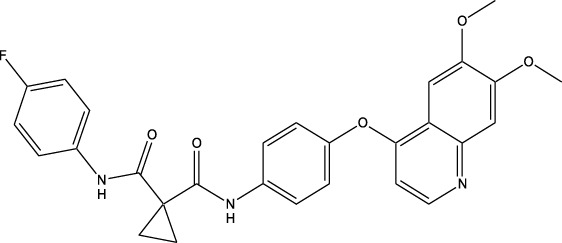	Various plants (fruits, vegetables)	MET, VEGFR, RET	Inhibits multiple receptor tyrosine kinases	Inhibits cell proliferation, reduces metastasis, used in medullary thyroid cancer and renal cell carcinoma	Sabbah et al. (2021)
Sorafenib (Nexavar)	Flavopiridol	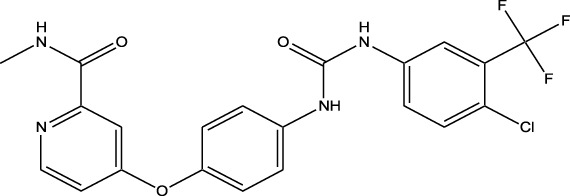	Plant (*Dysoxylum binectariferum*)	RAF kinases, VEGFR, PDGFR	Inhibits multiple kinases including RAF, VEGFR, and PDGFR	Induces apoptosis, inhibits angiogenesis, used in hepatocellular carcinoma, renal cell carcinoma, and thyroid cancer	Mohamed and El Kerdawy (2022)
Sunitinib (Sutent)	Quercetin	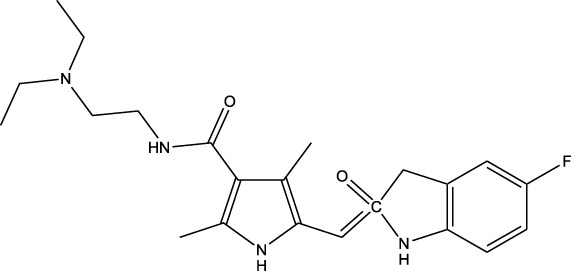	Various plants (fruits, vegetables)	VEGFR, PDGFR, c-Kit	Inhibits multiple receptor tyrosine kinases	Induces apoptosis, inhibits angiogenesis	Nagappa et al. (2020)

Abbreviations: FLT3: Fms-like Tyrosine Kinase 3; PKC: Protein Kinase C; c-Kit: Proto-Oncogene c-Kit; EGFR: epidermal growth factor receptor; mTOR: mechanistic Target of Rapamycin; VEGFR: vascular endothelial growth factor receptor; FGFR: fibroblast growth factor receptor; PDGFR: Platelet-Derived Growth Factor Receptor; MET: MET, Proto-Oncogene, Receptor Tyrosine Kinase; RET: rearranged during transfection.

### 4.7 Mechanisms of action of NKIs

Protein kinase inhibitors are categorized by the type of activity they display (Figure 2). Type I, II, and III kinase inhibitors were the initial classifications for small molecule protein kinase inhibitors (Dar and Shokat, 2011). Type II inhibitors attach themselves to conformations lack phosphate groups and are in a non-active site of the catalytic core of kinases (Liu and Gray, 2006). In contrast, type I kinase inhibitors attach to the ATP site when a kinase is in its active form, removing any conformation would otherwise be permissive for phosphotransferase (Baier and Szyszka, 2020). Typically, type I class are identified by a heterocyclic ring structure mimics the purine ring linked to the adenine of ATP to occupy the purine-similar binding site (Davis et al., 2011).

Initially, non-competitive or allosteric inhibitors were used to characterize type III inhibitors (Zucc et al., 2010). The allosteric inhibitors were subsequently divided into classes III and IV (Gavrin and Saiah, 2013). Type IV compounds are complementary to the narrow groove elsewhere in the phosphor-acceptor domain, whereas type III inhibitors are described as those target the narrow groove around the location where ATP is bound within the large and tiny lobes. Class III inhibitors work by inhibiting the phosphate donor in an uncompetitive or noncompetitive manner. This suggests this specific interaction between this molecule and the kinase cannot occur, even in elevated ATP levels. Substance binding sites, located outside of ATP binding pockets, are another setting in which type IV molecules may interact (Blanc et al., 2013). Thus, Initially, non-competitive or allosteric inhibitors were used to characterize type III inhibitors. Lastly, substances permanently attached themselves to the enzyme were called type V compounds. Unlike other medications target the cysteine located within the pocket where ATP is bound, the link forms between the cells and the kinase’s active site is permanent (Lamba and Ghosh, 2012). There are intrinsic distinctions between therapeutic compounds from these classes in terms of selectivity and safety. However, class I targets the ATP pocket throughout the kinome making them less selective. Type II drugs bind with the kinase’s inactive form and is less conserved throughout the kinome, they give higher selectivity (Kufareva and Abagyan, 2008).

### 4.8 Limitations and clinical gaps

Despite promising molecular and early clinical findings, several limitations hinder the widespread clinical adoption of natural kinase inhibitors. These include poor oral bioavailability, rapid metabolism, variability in formulation quality, and a lack of large-scale, lung cancer–specific randomized clinical trials. Most available clinical data originate from small cohort studies or trials conducted in heterogeneous solid tumor populations. Consequently, further well-designed clinical trials focusing on standardized formulations, optimized delivery systems (such as nanoformulations), and lung cancer–specific endpoints are required to validate the clinical efficacy and safety of NKIs.

## 5 Tyrosine kinases and their inhibitory effect

Certain tyrosine residues at protein targets are phosphorylated by a category of enzymes called tyrosine kinases using ATP as a catalyst (Thomson et al., 2023; Paul and Mukhopadhyay, 2004). Collectively, tyrosine kinases (TK) phosphorylate specific amino acids on substrate enzymes, altering the transmission of signals and, therefore, the functioning of cells. TKs trigger downstream signal transduction which may influence the development of cells, movement, distinction, and death. Through alterations or other processes, continuous activation as well as inhibition can lead to dysregulated signaling cascades, may result in malignancy and other illnesses (Jiao et al., 2018). Thus, by blocking these initial signals, TKIs can prevent the aberrant activation of the mutant or dysfunctional TKs. Human kinases have comparable three-dimensional structures while having different primary amino acid sequences, especially with regard to the pocket binds ATP in the active catalytic area. Usually conserved is the first amino acid sequence (ASP-Phe-Gly or DFG) of the flexible activation loop regulates access to the activation site (Tong and Seeliger, 2015).

TKs are classified as RTKs, non-receptor tyrosine kinases (NRTKs), and a small family of dual-specificity kinases (DSK) serine, phosphorylate tyrosine, and threonine residues. A single-pass transmembrane hydrophobic helix, a multidomain extracellular ligand for ligand particularity, and a cytoplasmic region containing a TK domain make up the structural configuration of the RTK. Both the N and C terminal ends of the kinase domain include regulatory sequences (Eshaq et al., 2024). ErbB, the insulin receptor (InsR) family, platelet-derived growth factor receptors (PDGFR) and the VEGFR receptor family EGFR and human epidermal growth factor receptor-2 (HER2) 137. The RTKs are enzymes with kinase activity in addition to being transmembrane receptors on the cell surface. NRTK proteins are found in the cytoplasm and have a great deal of structural variation. In addition to its kinase domain, the NRTK often features many signaling or protein-protein interaction domains, including the PH, SH2, and SH3 domains (Hubbard et al., 1998). Nine families make up NRTKs: Jak, Ack, Abl, Syk/Zap70, Tec, Fak, Csk, Fes/Fer, and Src; moreover, three families are not included in the list: Rak/Frk, Rlk/Txk, Brl/Sik and Srm. Mitogen-activated protein kinases (MEKs), mostly participate in the MAP pathways, are among the most widely known DSKs. The protein-serine/threonine kinase family contains a limited number of dual-specificity kinases, like MEK1 and MEK2 and catalyze the phosphorylation of both tyrosine and threonine in target proteins (Roskoski, 2012). Irreversible inhibition is produced by the kinase inhibitors’ propensity to covalently attach to and block the ATP site. Reversible kinase inhibitors can be categorized into four primary categories based on binding pocket validation and their DFG motif (Norman et al., 2012) (Table 4).

**TABLE 4:** Various binding modes of TKIs reveal structural diversity and mechanisms of action in targeted therapy. (Wu and Park, 2015).

Types	Binding modes
Type I inhibitors	Type I inhibitors bind competitively to the ATP-binding site of active TKs. In this interaction, the aspartate residue within the DFG motif is positioned toward the kinase’s catalytic site
Type II inhibitors	It binds to the ATP-binding site of inactive kinases, with the DFG motif extending beyond this site. Many type II inhibitors can also exploit regions near the ATP-binding site that would typically be inaccessible due to the outward rotation of the DFG motif
Type III inhibitors	There is no interaction with the ATP-binding pocket. Type III inhibitors exclusively bind to allosteric pockets adjacent to the ATP-binding site
Type IV inhibitors	Binds to allosteric sites located away from the ATP-binding pocket.
Type V inhibitors	Consists of compounds with many binding mechanisms

### 5.1 TK and their effects on lung cancer cells

TK has a role in the development and metastasis of cancer at several stages. TK signaling pathways often either prevent unchecked development or make people more sensitive to apoptotic triggers. These pathways of signaling are often altered biologically or genetically in cells with cancer to offer them an unfair edge. The signaling network fails as a result of these enzymes having a dominant oncoprotein status due to unusually strong TK signaling (Du and Lovly, 2018). Numerous disease processes include protein kinase mutations, dysregulation, and overexpression. Approximately 50% of human genes correspond to either cancer amplicons or disease loci, and one out of every 40 genomes codes for a protein kinase (Bhanumathy et al., 2021).

The FDA approved imatinib, a TKI, in 2001, sparking interest in protein kinase inhibitors. Due to TK’s vital involvement in cellular signaling, the use of TKIs has increased dramatically since the release of imatinib, especially in the treatment of cancer (Pophali and Patnaik, 2016). When the receptor is constitutively activated due to common mutations, like deletions in exon 19 (Del19) or the exon 21 substitution L858R, first-generation EGFR TKIs like gefitinib and erlotinib bind reversibly to the EGFR kinase domain, effectively blocking the receptor’s activity (Shah and Lester, 2020). These drugs inhibit autophosphorylation of the intracellular catalytic (kinase) region of the receptor by interacting with adenosine triphosphate (ATP) to limit EGFR signaling. This reduces the stimulation of intracellular signaling pathways (Spicer and Rudman, 2010). Gefitinib or erlotinib can be used as first-line therapy for NSCLC tumors have an EGFR mutation. However, most patients ultimately develop a resistance to these drugs (Fu et al., 2022).

### 5.2 Impact on cancer cell signaling pathways

The serine/threonine kinases ERK1 and ERK2 are part of the Ras-Raf-MEK-ERK MAP kinase signaling pathway and is crucial for regulating critical cellular functions like growth, division, and apoptosis. Many malignancies have the characteristic of dysregulation of this system and is frequently brought on by mutations in upstream elements like RAF and RAS. These mutations promote carcinogenic activity by constitutively activating the MAPK pathway. ERK1/2 targeting has become a viable treatment approach. Ulicixertinib and MK-8353 are two examples of small chemical inhibitors have demonstrated efficacy in blocking ERK1/2 activity and in turn disrupts cancer cell signaling and triggers apoptosis. These inhibitors may be able to overcome treatment resistance, especially in cancers are resistant to current RAF and MEK inhibitors (Roskoski, 2023).

ERK inhibition has important clinical consequences, especially in malignancies with RAF or RAS mutations. Although combinations of RAF and MEK inhibitors are the mainstay of current therapy, resistance to these medicines frequently develops, requiring other therapeutic choices. Since ERK inhibitors target a downstream MAPK pathway component is still active after RAF and MEK inhibition, they are a natural continuation of this treatment strategy. According to preliminary clinical studies, phosphorylation of ERK1/2 and its substrates can be efficiently reduced by ERK inhibitors, offering a dual mode of action may improve therapy effectiveness (Song et al., 2023).

### 5.3 Dual and multi-target inhibitors

Currently, there are two competing approaches to developing drugs with many targets. The first tactic is combination drug treatment and works by combining several medications operate on several targets to provide a synergistic or cumulative effect. In several instances, combination treatment has proven to be effective. Preclinical data has shown they promote apoptosis and delay the onset of resistance to serine/threonine protein kinase B-Raf (Flaherty et al., 2012). Phase III clinical studies have demonstrated the potential of combination treatment utilizing both MEK inhibitor (cobimetinib) and RAF inhibitor (vemurafenib) in treating BRAF-mutated melanoma (Larkin et al., 2014). The combination treatment of palbociclib and letrozole for advanced breast cancer is another instance of effective combined therapy (Raghavendra et al., 2018). Treatment for SCLC has included the effective use of a mixture of medicines (doxorubicin, vincristine, topotecan, and cyclophosphamide) inhibit various pathways (Von Pawel et al., 1999). Adriamycin and cyclophosphamide are combined among popular regimens, known as “AC.” When docetaxel is added, the regimen is referred to as “AC-T″ and is used to treat breast cancer all over the globe. The second tactic involves creating medications target numerous oncogenic pathways simultaneously to effectively inhibit them all (Keith et al., 2005).

The process of developing a single drug can operate on two or more sites at once is known as multi-targeting therapy. Lenvima (lenvatinib), for instance, is a receptor TKI the US FDA has licensed. Vascular endothelial growth factor (VEGF) receptors VEGFR3, VEGFR2 and VEGFR1 are inhibited in their kinase activity (Fala, 2015). Cabozantinib, commonly referred to as cabometyx, is a small molecule dual-targeting inhibitor of VEGFR2 and c-Met TKs has been approved by the FDA. It has been shown to inhibit angiogenesis, spread, and tumor growth (Santoni et al., 2021).

### 5.4 Preclinical studies

**FIGURE 8:**
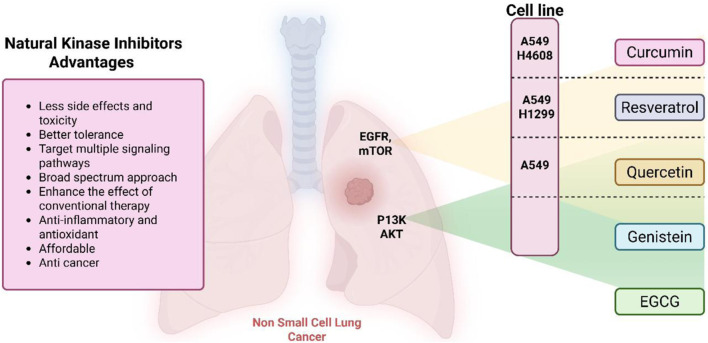
Highlights the benefits of natural kinase inhibitors and their targeted effects on specific lung cancer cell lines, particularly in Small Cell Lung Cancer (SCLC). Natural compounds such as Curcumin, Resveratrol, Quercetin, Genistein, and EGCG are shown to exert anti-cancer effects on various lung cancer cell lines including A549, H4608, H1299, and others. These compounds modulate key pathways such as EGFR, mTOR, PI3K, and AKT, contributing to cancer cell growth inhibition. Their advantages include reduced toxicity, better tolerance, multi-targeted pathway inhibition, anti-inflammatory and antioxidant effects, and affordability, making them promising adjuncts or alternatives to conventional lung cancer therapies. Created in https://BioRender.com Abbreviations: AKT, protein kinase B; EGFR, epidermal growth factor receptor; PI3K, phosphoinositide 3-kinase; SCLC, small cell lung cancer; mTOR, mechanistic target of rapamycin.

An enormous assortment of natural goods has demonstrated significant efficacy in targeting different signaling channels to provide tumor-inhibiting and antiproliferative effects, particularly in preclinical studies (Figure 8). *In vitro* research has shown curcumin can cause apoptosis and cell cycle arrest in NSCLC cell lines, like A549 and H460, showcasing its viability as a treatment, as mentioned in Table 5 (Islam et al., 2024). Similarly, one naturally occurring substance in grapes is resveratrol exhibits anti-cancer properties by modulating the same pathways, leading to reduced tumor growth and enhanced apoptosis in NSCLC models. Preclinical research has demonstrated resveratrol can inhibit the growth of A549 and H1299 cell lines and has anti-angiogenic effects, further supporting its role in cancer therapy (Yousef et al., 2017). Quercetin has been shown to inhibit various kinases, including EGFR and mTOR. *In vivo* studies with quercetin have demonstrated significant tumor growth inhibition and apoptosis in A-549 nude mouse xenografts models (Zheng et al., 2012). Genistein, derived from soybeans, has shown efficacy in inhibiting tumor growth in preclinical models by targeting the PI3K/AKT pathway (Sharifi-Rad et al., 2021), while EGCG (epigallocatechin gallate) from green tea has shown pro-oxidative activities inhibit tumorigenesis in animal models of lung cancer xenografts (Table 5).

**TABLE 5:** Plant-derived kinase inhibitors offer promising, natural alternatives for targeted cancer therapy and drug development.

Compound	Chemical Structure	Source	Kinases Inhibited	Mechanisms of Action	Anti-Cancer Effects	References
Curcumin	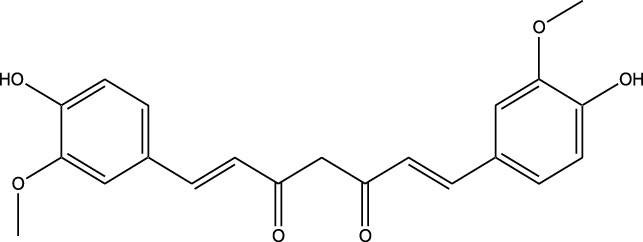	Turmeric (*Curcuma longa*)	EGFR, AKT, JAK2, PKC	Inhibits NF-κB, MAPK, and PI3K/AKT pathways	Induces apoptosis, inhibits proliferation, suppresses angiogenesis, and metastasis	Ashrafizadeh et al. (2020)
Resveratrol	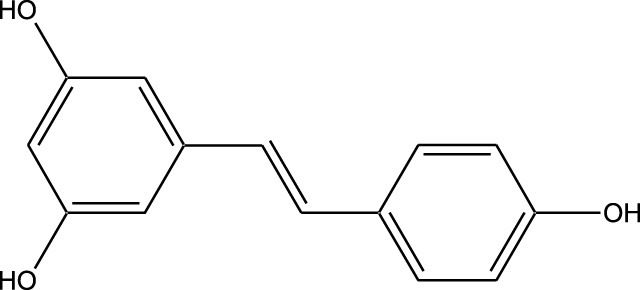	Grapes, berries, peanuts, red wine	PI3K, AKT	Inhibits PI3K/AKT and NF-κB pathways, modulates MAPK	Induces apoptosis, inhibits proliferation, suppresses tumour growth and metastasis	Ren et al. (2021)
Quercetin	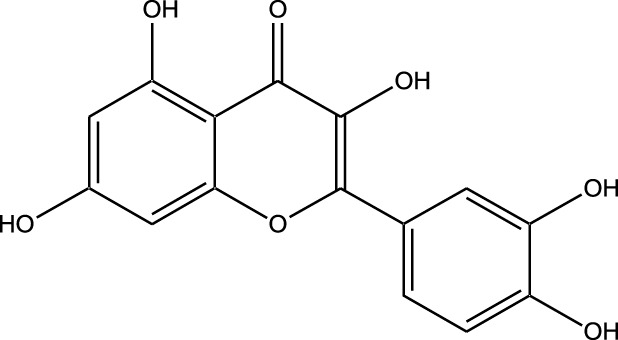	Onions, apples, berries	PI3K, Src, EGFR	Inhibits NF-κB and MAPK pathways	Induces apoptosis, inhibits proliferation, reduces inflammation and angiogenesis	Ay et al. (2021)
Genistein	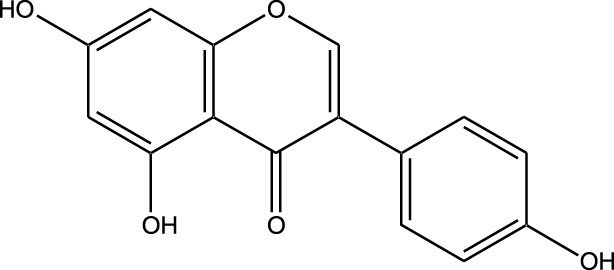	Soybeans, soy products	EGFR, HER2, PI3K	Inhibits tyrosine kinases, modulates MAPK and PI3K/AKT pathways	Inhibits tumour growth, induces apoptosis, reduces metastasis	Sharifi-Rad et al. (2021)
Apigenin	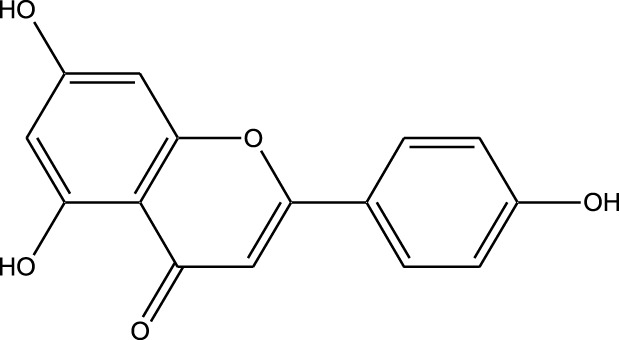	Parsley, celery, chamomile	EGFR, PI3K	Inhibits NF-κB and MAPK pathways	Induces apoptosis, inhibits proliferation, suppresses tumor growth	Patel and Singh (2022)
Honokiol	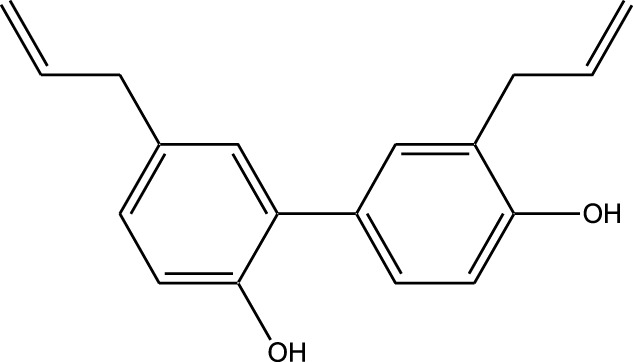	*Magnolia* species	EGFR, HER2	Inhibits PI3K/AKT and MAPK pathways	Induces apoptosis, inhibits proliferation, reduces metastasis	Dai et al. (2023)
Andrographolide	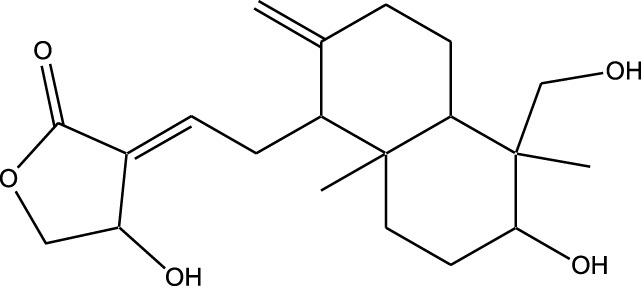	*Andrographis paniculata*	EGFR, HER2	Inhibits NF-κB and JAK/STAT pathways	Induces apoptosis, inhibits proliferation, reduces inflammation and metastasis	Cai et al. (2022)
Berberine	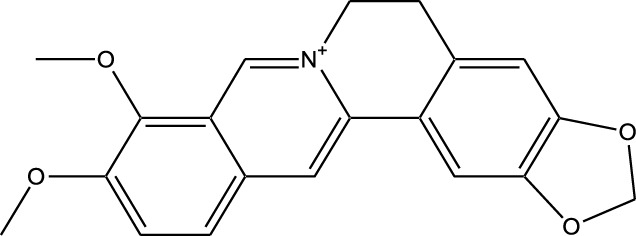	Berberis species	AMPK, MAPK, PI3K	Modulates PI3K/AKT pathway	Induces apoptosis, inhibits proliferation, suppresses tumor growth	Han et al. (2023)
Epigallocatechin gallate	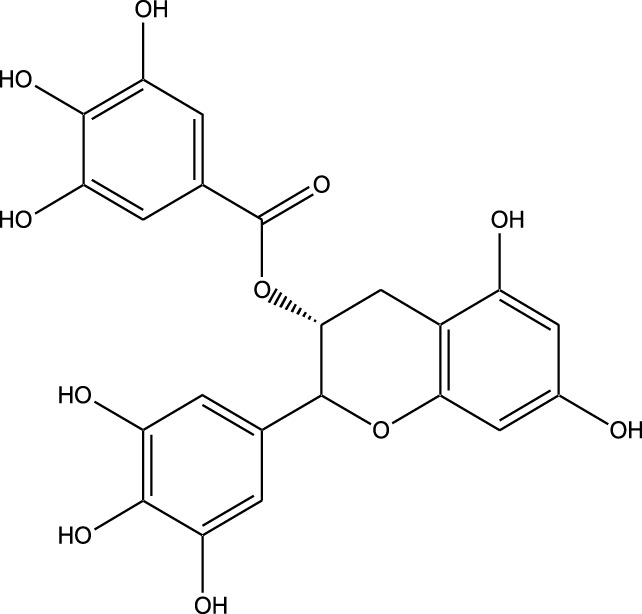	Green tea (*Camellia sinensis*)	EGFR, HER2	Inhibits PI3K/AKT and MAPK pathways, modulates MMP activity	Induces apoptosis, inhibits proliferation, suppresses angiogenesis and metastasis in various cancers	Liao et al. (2020)
Baicalein	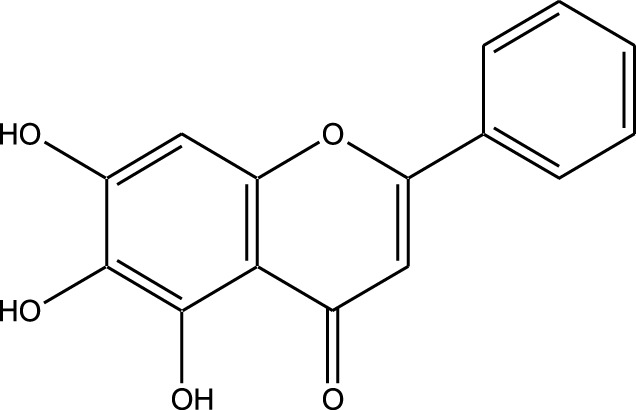	*Scutellaria baicalensis*	MEK, ERK	Inhibits MEK/ERK pathway	Induces apoptosis, inhibits proliferation, reduces metastasis	Guo et al. (2022)
Fisetin	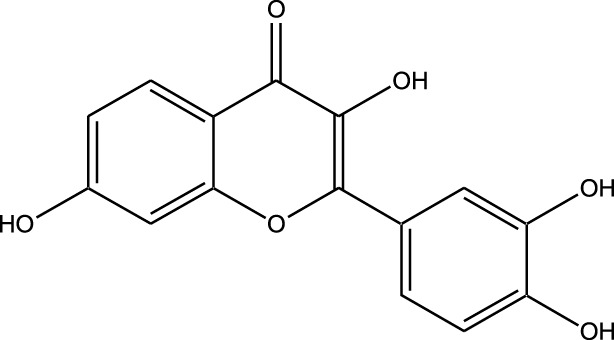	Strawberries, apples, persimmons	mTOR, CDKs	Inhibits mTOR and CDK pathways	Induces apoptosis, inhibits proliferation, reduces tumor growth	Jiang et al. (2024b)
Luteolin	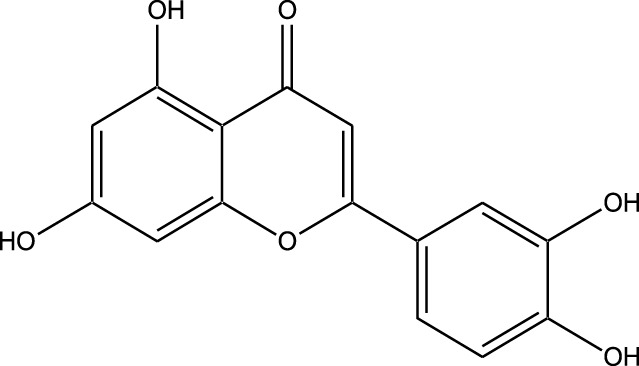	Peppers, celery, thyme	IGF-1R, PI3K, Akt	Inhibits PI3K/Akt pathway, modulates IGF-1R	Induces apoptosis, inhibits proliferation, reduces inflammation	Choi et al. (2024)
Kaempferol	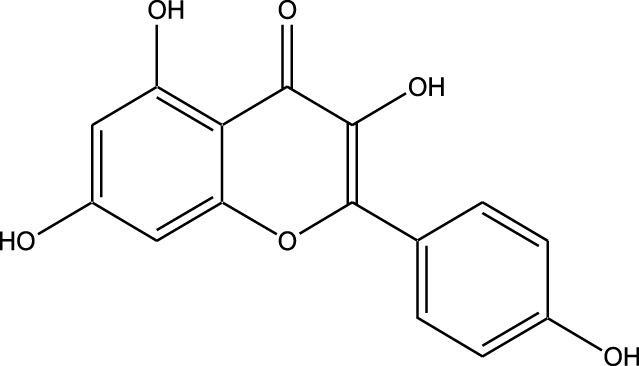	Tea, broccoli, grapefruit	Src, MEK, ERK	Inhibits Src, MEK/ERK pathways	Induces apoptosis, inhibits proliferation, reduces angiogenesis	Sharma et al. (2022b)
Sulforaphane	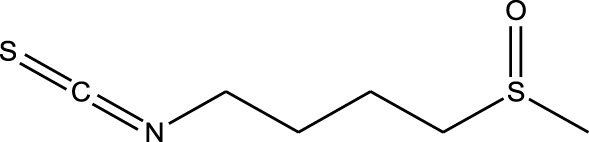	Broccoli, Brussels sprouts	NF-κB, Akt	Inhibits NF-κB and Akt pathways	Induces apoptosis, inhibits proliferation, reduces tumor growth	Múnera-Rodríguez et al. (2024)
Chrysin	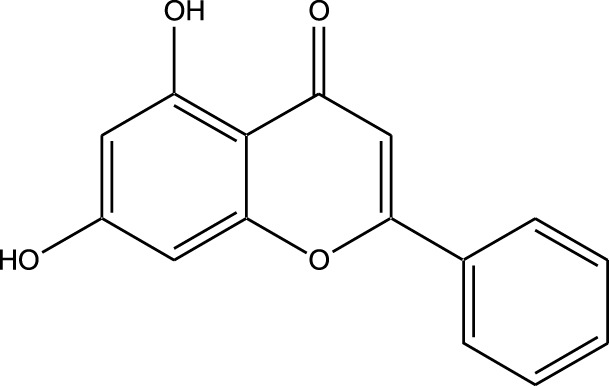	Honey, propolis, passion flowers	PI3K, Akt	Inhibits PI3K/Akt pathway	Induces apoptosis, inhibits proliferation	Li et al. (2022b)
Emodin	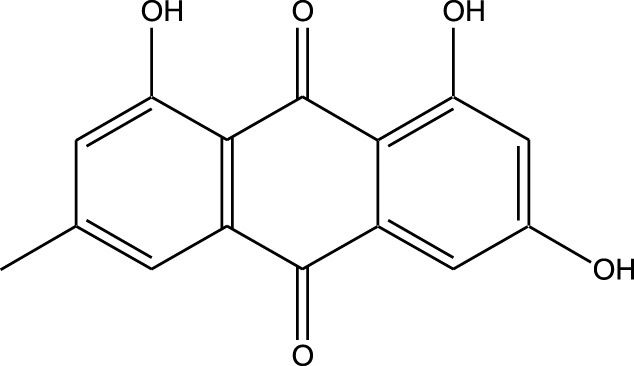	Rhubarb, aloe vera	EGFR, HER2, PI3K	Inhibits EGFR and HER2 kinases, modulates PI3K/AKT pathway	Induces apoptosis, inhibits proliferation, reduces metastasis	Zhang et al. (2024b)
Withaferin A	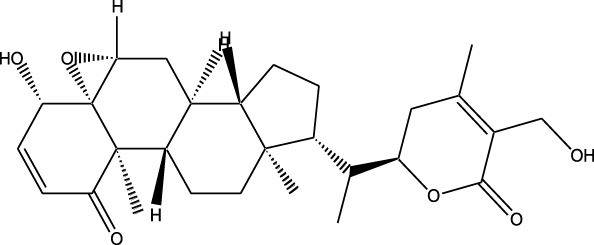	*Withania somnifera* (Ashwagandha)	Akt, NF-κB, JAK/STAT	Inhibits Akt and NF-κB pathways, modulates JAK/STAT signaling	Induces apoptosis, inhibits proliferation, reduces inflammation and metastasis	Yin et al. (2021)
Hesperidin	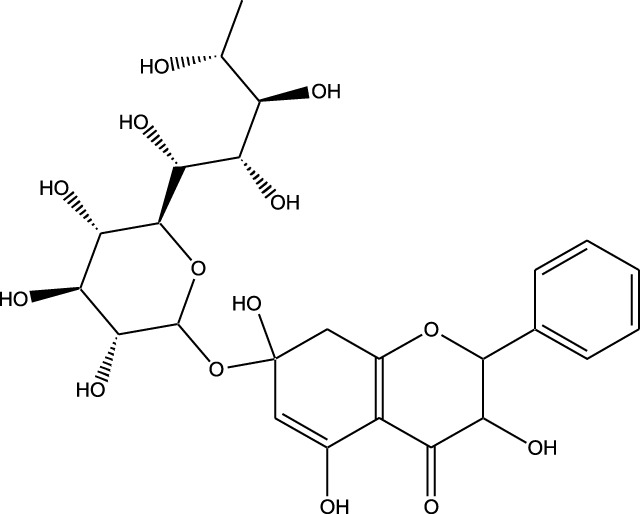	Citrus fruits	PI3K, Akt	Inhibits PI3K/Akt pathway	Induces apoptosis, inhibits proliferation, reduces inflammation	Huang et al. (2020)
Silibinin	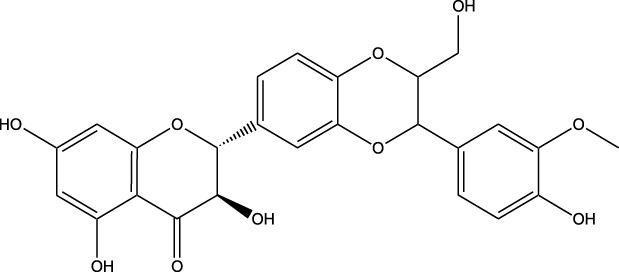	Milk thistle (*Silybum marianum*)	EGFR, HER2, PI3K, Akt	Inhibits EGFR and HER2 kinases, modulates PI3K/Akt pathway	Induces apoptosis, inhibits proliferation, suppresses angiogenesis and metastasis	Wang (2025)
Ellagic Acid	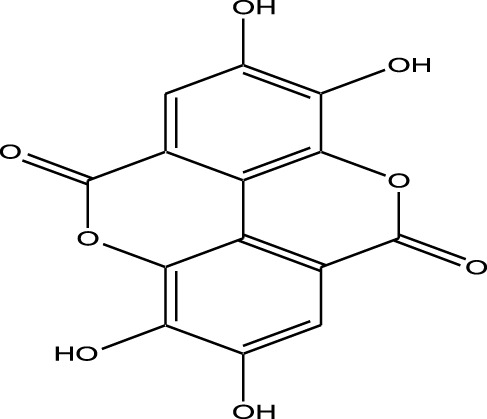	Pomegranates, berries	PI3K, Akt	Inhibits PI3K/Akt pathway	Induces apoptosis, inhibits proliferation, reduces inflammation and angiogenesis	Lu et al. (2023)
Lycopene	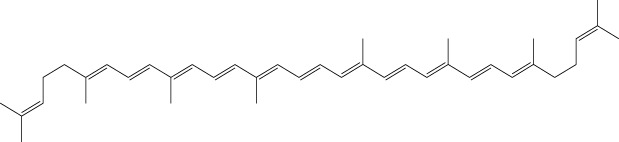	Tomatoes, watermelon	IGF-1R, PI3K, Akt	Inhibits IGF-1R, modulates PI3K/Akt pathway	Induces apoptosis, inhibits proliferation, reduces oxidative stress and inflammation	Tao et al. (2021)
Capsaicin	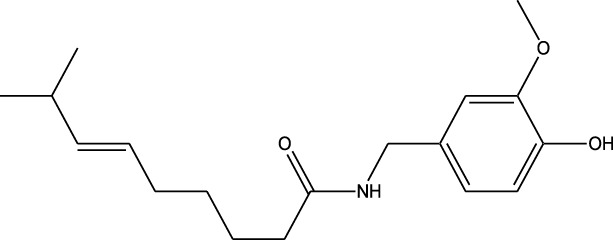	Chili peppers	EGFR, HER2, PI3K	Inhibits EGFR and HER2 kinases, modulates PI3K/Akt pathway	Induces apoptosis, inhibits proliferation, reduces inflammation and metastasis	Radhakrishna (2024)
Pterostilbene	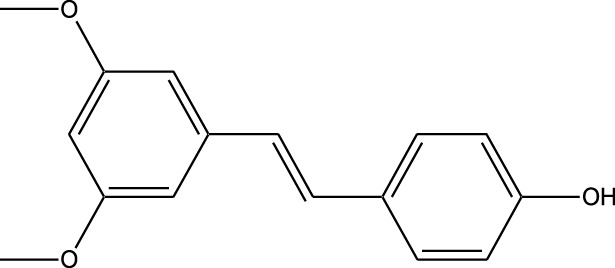	Blueberries, grapes	PI3K, Akt, AMPK	Inhibits PI3K/Akt and AMPK pathways	Induces apoptosis, inhibits proliferation, reduces inflammation and metastasis	Shen et al. (2023)
Thymoquinone	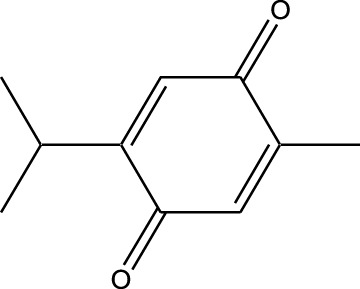	Black cumin seeds (*Nigella sativa*)	PI3K, Akt, JAK/STAT	Inhibits PI3K/Akt and JAK/STAT pathways	Induces apoptosis, inhibits proliferation, reduces inflammation and oxidative stress	Sadeghi et al. (2023)
Betulinic Acid	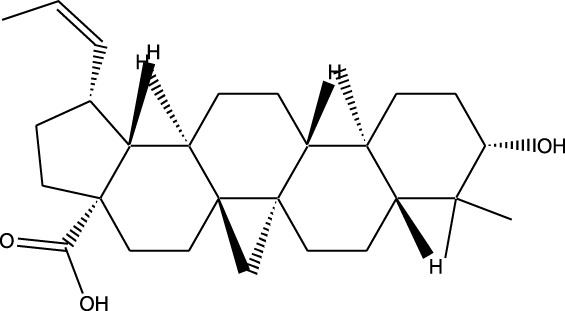	Birch bark	PI3K, Akt, MAPK	Inhibits PI3K/Akt and MAPK pathways	Induces apoptosis, inhibits proliferation, reduces inflammation and metastasis	Huang et al. (2025)
Ursolic Acid	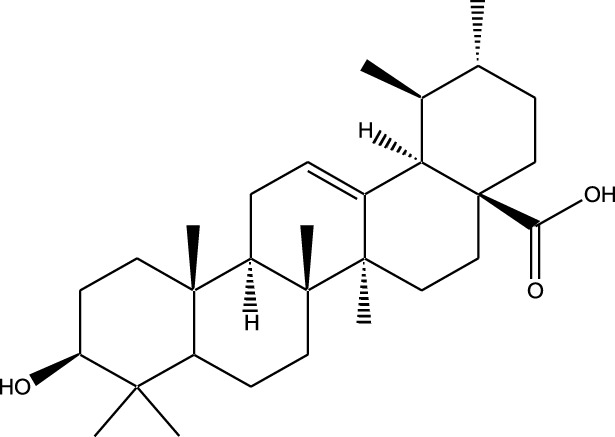	Apples, rosemary, holy basil	PI3K, Akt, NF-κB	Inhibits PI3K/Akt and NF-κB pathways	Induces apoptosis, inhibits proliferation, reduces inflammation and angiogenesis	Wang et al. (2020)
Isoliquiritigenin	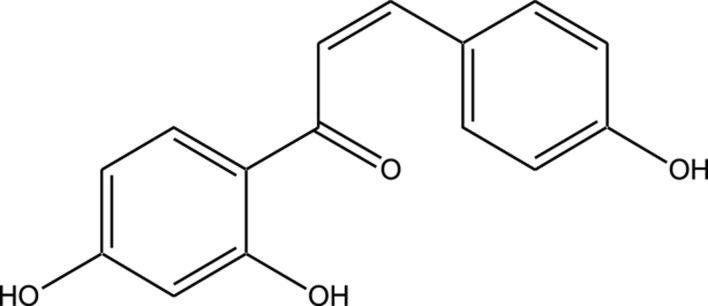	Licorice (*Glycyrrhiza glabra*)	PI3K, Akt, ERK	Inhibits PI3K/Akt and ERK pathways	Induces apoptosis, inhibits proliferation, reduces inflammation and metastasis	Ganesan et al. (2024)

Abbreviations: EGFR: epidermal growth factor receptor; AKT: Protein Kinase B; JAK2: Janus Kinase 2; PKC: Protein Kinase C; PI3K: Phosphoinositide 3-Kinase; NF-κB: Nuclear Factor Kappa-Light-Chain-Enhancer of Activated B Cells; MAPK: Mitogen-Activated Protein Kinase; HER2: Human Epidermal Growth Factor Receptor 2; HDACs: Histone Deacetylases; mTOR: mechanistic Target of Rapamycin; CDKs: Cyclin-Dependent Kinases; IGF-1R: Insulin-like Growth Factor 1 Receptor; Src: Sarcoma (Proto-Oncogene Tyrosine-Protein Kinase); MEK: Mitogen-Activated Protein Kinase; ERK: Extracellular Signal-Regulated Kinase; AMPK: AMP-Activated Protein Kinase; MMP: matrix metalloproteinase.

### 5.5 Advantages and cell line targets of natural kinase inhibitors in non-small cell lung cancer (NSCLC)

### 5.6 Clinical efficacy and safety studies

The effectiveness and safety of osimertinib as adjuvant treatment in patients with fully resected, EGFR-mutated, stage IB to NSCLC were assessed in the most current trial, a phase III, double-blind, placebo-controlled study carried out by Tsuboi et al., in 2023 where 682 participants were randomly assigned to receive either osimertinib or a placebo for a maximum of 3 years or until the criteria for discontinuation were met. Disease-free survival (DFS), as determined by researchers, was the trial’s main result. The SF-36 v2 summary scores (Mental Component Summary and Physical Component Summary) in the stage II to IIIA population were used to measure the secondary outcomes and included time to deterioration (TTD) and overall survival (OS) in health-related quality of life (HRQoL). The study population in this international phase III trial was arranged based on the stage of the sickness (IB, II, or IIIA), EGFR mutation type (exon 19 deletion or L858R), and race (Asian or non-Asian), and was evenly distributed between patients with mutations substitute exon 21 (L858R) and deletions of exon 19. The trial demonstrated a significant improvement in DFS and OS with Osimertinib compared to placebo, with a safety profile consistent with its known adverse event profile. The findings back up the use of osimertinib as a new standard of therapy for patients with stage IB to IIIA NSCLC has been fully resected and has an EGFR mutation. Osimertinib’s safety profile after prolonged follow-up was in line with the primary analysis of the trial’s finding (Tsuboi et al., 2023).

A different investigation by Wolf et al. was a multiple-cohort, prospective, multinational, open-label, phase 2 trial to assess the safety and effectiveness of capmatinib in patients with advanced NSCLC who had a MET exon 14 skipping mutation or MET amplification. The main outcome was the overall response (full or partial) and included 364 individuals. A total of 69 NSCLC patients who had previously had one or two lines of therapy and 28 patients who had never had treatment showed an overall response; the median response length was 9.7 months for the former group and 12.6 months for the latter. These patients had MET exon 14 skipping mutations. This investigation validated Capmatinib’s established safety profile. With dosage modifications, most side effects were predicted, grade 1 or 2, and reversible (Wolf et al., 2020).

According to Solomon et al. patients with advanced NSCLC was histologically or cytologically proven to be ALK-positive or ROS1-positive were assessed in a phase 2 trial. The main outcomes were intracranial tumor response and objective tumor response and was characterized as a verified full or partial response. With an ECOG performance rating of 0 or 1, the majority of patients were Caucasian or Asian in ethnicity. Thirty-six ROS1-positive patients had also had chemotherapy, and over A majority of patients with ALK positivity had received at least one ALK TKI therapy before. In line with its extensive CNS penetration and broad coverage of ALK mutations, lorlatinib demonstrated significant total and intracranial activity in patients with ALK-positive non-small cell lung cancer (NSCLC) who had not before been treated, as well as patients who advanced while using Crizotinib, a second-generation ALK TKI, or after taking up to three ALK TKIs in the past. As a result, lorlatinib may be a useful first- or second-line treatment for patients with NSCLC is positive for ALK (Solomon et al., 2018).

The clinical discussion of synthetic kinase inhibitors in this review is intended to illustrate validated kinase-targeting paradigms and resistance mechanisms. However, the core emphasis remains on natural kinase inhibitors as complementary and translationally relevant agents. Future clinical trials specifically designed to evaluate natural compounds—alone or in combination with standard therapies are essential to establish their definitive clinical utility in lung cancer.

### 5.7 Case studies and outcomes

A 75-year-old woman who has never smoked and has non-small cell lung cancer (NSCLC) with a MET exon 14 skipping mutation is described in a case report. A MET D1228H mutation caused the patient to gain resistance after initially responding favorably to capmatinib therapy, a type IB MET-TKI. Remarkably, the patient had a distinct response to savolitinib, another type Ib MET-TKI, in the second line, with a 19-month response length. But after more metastatic lesions developed, a re-biopsy showed the MET D1228H variation had been lost and the newly identified MET p. Y1230N variation had been found. The case report observed three significant findings: savolitinib effectiveness against the D1228H mutation, the distinct resistance mechanisms observed between type Ib MET-TKIs, and the necessity for further research using patient-derived pre-clinical models to enhance treatment strategies for MET-driven NSCLC (Li X. et al., 2024).

Another case of an 84-year-old man presented with fatigue and dizziness, leading to a diagnosis of Philadelphia chromosome-positive (Ph+) acute lymphoblastic leukemia (ALL). Concurrently, imaging revealed a lung mass and was later confirmed as lung adenocarcinoma (LUAD) with an EGFR exon 19 mutation. Both conditions are linked to abnormal activation of the TK pathway. The patient was initially treated with the TKI flumatinib for ALL and resulted in significant improvement and normalization of blood tests within a month. Subsequently, the patient’s lung condition deteriorated, prompting treatment with pemetrexed and cisplatin for pleural effusion. Eventually, he was switched to EGFR-TKI oxertinib, leading to a marked reduction in lung lesions over 8 months. Remarkably, the patient experienced no side effects during the dual TKI therapy. This case highlights the rare synchronous occurrence of Ph + ALL and EGFR mutant LUAD, suggesting potential shared pathways in their pathogenesis and the feasibility of treating both malignancies with targeted therapies effectively. Further research is warranted to explore the interactions between these diseases and their treatments (Zhang Q. et al., 2024).

Another case report discusses the efficacy of afatinib highlighting two cases. Afatinib a second-generation EGFR TKI, in treating two patients with advanced NSCLC complicated by leptomeningeal metastases (LM) and resistance to first- and third-generation TKIs. Case 1 involves a 43-year-old man initially diagnosed with stage IIIA NSCLC with an EGFR exon 19 deletion. After prolonged treatment with icotinib and later osimertinib, he developed LM, characterized by tumor cells in cerebrospinal fluid (CSF). Following the detection of various mutations, including EGFR L858R, he was treated with afatinib and intrathecal chemotherapy, resulting in significant clinical improvement and a performance status (PS) score of 1 after 10 months. Case 2 describes a 50-year-old man diagnosed with NSCLC harboring the EGFR L858R mutation. After treatment with icotinib and osimertinib, he also developed LM. Despite limited changes in his mutation profile, afatinib was administered, leading to stable disease for 11 months. Both cases suggest afatinib may effectively overcome resistance in NSCLC patients with complex EGFR mutations and LM, offering a viable treatment option when other TKIs fail (He et al., 2024).

## 6 Challenges future directions

Numerous preclinical research and *in vitro* investigations have demonstrated the potent anticancer effects of natural substances; however, the low oral bioavailability, instability, and low aqueous solubility of natural polyphenolic compounds have complicated the translation of these results into human trials. These drugs frequently experience substantial metabolism in the liver and small intestine and leaves the systemic circulation with very low quantities of active forms (Chunarkar-Patil et al., 2024). Phase II enzymes, for example, have the ability to modify flavonoids extensively, producing derivatives with unknown pharmacological characteristics. Because the concentrations of parent compounds in human plasma are far lower than those have been demonstrated in preclinical studies to be pharmacologically efficacious, novel drug delivery methods must be created to maximize the therapeutic uses of these compounds (Suraweera et al., 2020).

With the use of nano-sized carriers, nanotechnology has shown promise in addressing these concerns by delivering active molecules directly to target tissues, like cancer. Through ligand-receptor interactions, these nanoparticles can actively engage with tumor cells or passively accumulate in tumors as a result of aberrant angiogenesis. Systemic toxicity is reduced, and bioavailability is enhanced by this tailored administration. The creation of nanoformulations is not without its difficulties, though; these include problems with distribution, production, and possible toxicity. Particle size and surface properties are two important factors affect pharmacokinetics and biodistribution. Furthermore, immunological responses to nanoparticles may be triggered and might result in consequences including hypersensitivity reactions (Yao et al., 2020). Numerous studies have shown how well nanotechnology works to increase the bioavailability of particular natural substances. Quercetin nanoparticles, for example, have demonstrated improved solubility and multifunctional benefits in the treatment of illnesses associated with amyloid (Pinheiro et al., 2021). In a similar vein, resveratrol stabilized against pre-systemic metabolism and enhanced oral bioavailability when encapsulated in casein nanoparticles (Alfei, 2020). Other examples are lipid nanoconstructs encapsulated in curcumin and greatly increases solubility, and honokiol nanoparticles and dramatically boost solubility and bioavailability (Jain et al., 2024b). Furthermore, plumbagin nanoemulsions have demonstrated increased anticancer activity against prostate cancers. These developments demonstrate how nanotechnology may be used to get around the drawbacks of natural substances and increase the effectiveness of their therapy for treating cancer (Jain et al., 2024c).

## 7 Conclusion

This review underscores natural kinase inhibitors (NKIs) as a distinctive, multi-targeted class capable of modulating core oncogenic signalling in lung cancer—including EGFR, PI3K/AKT/mTOR, and MAPK while offering favorable tolerability and a reduced propensity for resistance. Rather than reiterating mechanistic detail, the central insight is translational: representative NKIs (e.g., curcumin, resveratrol, quercetin, EGCG, genistein, berberine) converge on kinase-driven survival and proliferation circuits and, through polypharmacology, counter tumour heterogeneity, signalling plasticity, and microenvironmental crosstalk. These attributes align with the clinical need for safer, durable, and cost-effective regimens that complement or enhance existing standards of care.

Crucially, the therapeutic promise of NKIs is magnified when integrated into a NKIs, immunotherapy and nanomedicine framework. NKIs can reprogramme the tumour microenvironment attenuating pro-inflammatory and immune-evasive signals (e.g., NF-κB, JAK/STAT) to potentiate checkpoint blockade, while nanotechnology enables precision delivery that elevates intratumoural exposure, improves pharmacokinetics, and minimises off-target toxicity. This synergy supports combination strategies that simultaneously (i) dampen oncogenic kinase activity, (ii) amplify antitumour immunity, and (iii) solve bioavailability and selectivity constraints through targeted carriers. Taken together, the triad offers a coherent path to superior efficacy and durability *versus* single-modality therapy.

Looking forward, four priority tracks should structure NKI translation. (1) Clinical validation and large-scale trials: multi-centre, randomised studies testing NKI-based combinations against current standards, with endpoints encompassing overall survival, progression-free survival, intracranial control, patient-reported outcomes, and cost-effectiveness; pragmatic designs in real-world populations to capture generalisability. (2) Optimisation of combination strategies: rational scheduling and dosing with immune checkpoint inhibitors and tyrosine-kinase inhibitors; biomarker-guided selection (genomic drivers, phosphoproteomic signatures, immune phenotypes); adaptive trial frameworks to refine responders and de-risk toxicity. (3) Mechanistic studies to clarify molecular pathways: deep phospho-proteomics, single-cell multi-omics, and spatial transcriptomics to map target engagement, network rewiring, and resistance nodes (bypass activation, EMT, metabolic shifts); systems pharmacology to quantify synergy and identify actionable feedback loops. (4) Development of targeted delivery using nanotechnology: tumour-selective nanoparticles (ligand-directed, EPR-enhanced), stimuli-responsive release (pH, redox, enzymatic triggers), hybrid lipid–polymer platforms, and inhalable formulations for lung-focused deposition; rigorous characterisation of biodistribution, manufacturability, and immunocompatibility.

By bridging natural-product pharmacology with immuno-oncology and nanotechnology, NKIs advance from promising adjuncts to credible pillars of precision medicine. If pursued with disciplined clinical science and delivery innovation, they can reshape therapeutic paradigms offering safer, smarter, and more sustainable control of lung cancer. In sum, NKIs stand as promising candidates for future oncological innovations, defining an emerging frontier where multi-target kinase modulation, immune activation, and engineered delivery coalesce to achieve meaningful, durable patient benefit.
